# Proteomic Characterization of 1000 Human and Murine Neutrophils Freshly Isolated From Blood and Sites of Sterile Inflammation

**DOI:** 10.1016/j.mcpro.2024.100858

**Published:** 2024-10-11

**Authors:** Susmita Ghosh, Ali Ata Tuz, Martin Stenzel, Vikramjeet Singh, Mathis Richter, Oliver Soehnlein, Emanuel Lange, Robert Heyer, Zülal Cibir, Alexander Beer, Marcel Jung, Dennis Nagel, Dirk M. Hermann, Anja Hasenberg, Anika Grüneboom, Albert Sickmann, Matthias Gunzer

**Affiliations:** 1 Leibniz-Institut für Analytische Wissenschaften – ISAS – e.V., Dortmund, Germany; 2 Institute for Experimental Immunology and Imaging, University Hospital, University of Duisburg-Essen, Essen, Germany; 3 Institute for Experimental Pathology, University of Münster, Münster, Germany; 4 Multidimensional Omics Analyses Group, Faculty of Technology, Bielefeld University, Universitätsstraße 25, Bielefeld, Germany; 5 Department of Neurology, University Hospital Essen, University of Duisburg-Essen, Essen, Germany; 6 Medizinisches Proteom-Center, Ruhr-Universität Bochum, Bochum, Germany; 7 Department of Chemistry, College of Physical Sciences, University of Aberdeen, Aberdeen, UK

**Keywords:** neutrophils, low input proteomics, circulation, inflammation, stroke, transmigration

## Abstract

Neutrophils are indispensable for defense against pathogens. Injured tissue-infiltrated neutrophils can establish a niche of chronic inflammation and promote degeneration. Studies investigated transcriptome of single-infiltrated neutrophils which could misinterpret molecular states of these post mitotic cells. However, neutrophil proteome characterization has been challenging due to low harvests from affected tissues. Here, we present a workflow to obtain proteome of 1000 murine and human tissue-infiltrated neutrophils. We generated spectral libraries containing ∼6200 mouse and ∼5300 human proteins from circulating neutrophils. 4800 mouse and 3400 human proteins were recovered from 1000 cells with 10^2^-10^8^ copies/cell. Neutrophils from stroke-affected mouse brains adapted to the glucose-deprived environment with increased mitochondrial activity and ROS-production, while cells invading inflamed human oral cavities increased phagocytosis and granule release. We provide an extensive protein repository for resting human and mouse neutrophils, identify proteins lost in low input samples, thus enabling the proteomic characterization of limited tissue-infiltrated neutrophils.

## Highlights

• Proteome analysis of as few as 1000 neutrophils.
• Spectral library of human and mouse resting circulatory neutrophils.
• A webserver for neutrophil protein copy numbers in human and mouse.
• Species specific similarity and divergence of neutrophil proteome.
• Alteration in neutrophil proteome under various inflammatory contexts.

## Graphical Abstract

**Figure:**
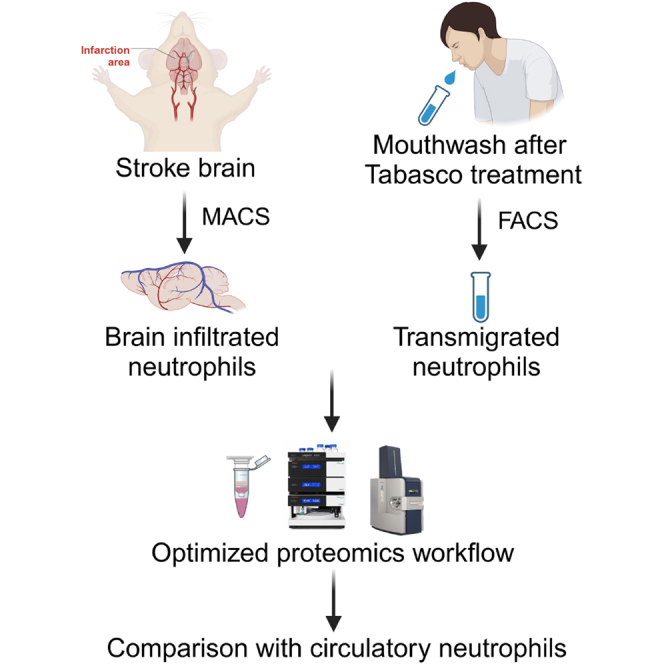

## In Brief

Neutrophils, even in small numbers (~1000 cells) can infiltrate inflammatory sites and make diseases worse. Until now no study performed the proteome analysis of the scant populations of neutrophils from a single inflammatory site without pooling. This study provides an optimized sample preparation and LC-MS-based proteomics workflow which enables profiling of neutrophils isolated from a single inflammatory site in an experimental animal or human donor with substantial proteome coverage.

Neutrophils are the most abundant circulating immune cells in the human blood and central for fighting microbial infections (1). However, their influence extends well beyond innate immune defense, significantly impacting various physiological and pathological processes such as tumor growth, progression of chronic inflammation, as well as immunosuppression (2, 3, 4). Neutrophils are also known to respond rapidly and extensively to sterile inflammation, thereby exhibiting functional heterogeneity in the transition from acute to chronic inflammation (5, 6). These cells have been phenotypically characterized with antibody-based assays based on surface markers (3). Recently, several studies employing single-cell transcriptome analyses have been performed (7), especially focusing on cells from the inflamed tissues (8, 9). It has, however, been shown that transcriptome data of myeloid cells do not necessarily match with proteome data in timing and/or extent of gene/protein expression (10). Thus, while a transcriptome shows more the general possibilities of cells for molecular composition, a proteome depicts the real molecular content, emphasizing proteins as the key drivers of biological functions. In light of the findings on RNA–protein mismatches (10), this appears to be especially important for neutrophils, which are post-mitotic cells. Hence, a comprehensive characterization of neutrophils using proteomics would provide different insights into the molecular intricacies underlying the functional dynamics under pathophysiological conditions. In healthy humans, neutrophils make up 60% of the white cells in blood and in mice, this number is at 10 to 20% (11, 12). However, their quantity at inflammatory sites, especially in chronic inflammation or steady-state infiltration is typically much lower, often involving 1000 cells or even less (13, 14). Especially also when sorting rare populations of neutrophils from tumors, availability of material becomes a serious issue (15). Thus, the limited cell numbers poses a considerable challenge for proteome profiling using conventional approaches. Recent proteomics advancements have enabled adequate proteome coverage from low inputs using methods like SP3 (16), micro FASP (17), in-cell digestion (18), even down to single mammalian cells using nanoPOTS (19), single pot (20), or ProteoCHIP (21). However, most of these protocols are optimized for large-size immortalized cell lines, that surpass the volumes of primary immune cells several fold (*e.g.* HeLa cells have a volume of 3700 μm (3, 22)), while human neutrophils have a volume of ∼300 μm^3^ in the microcirculation (23)). Hence, despite many technical advancements in proteomics, there is currently a lack of technologies for proteomic analyses of low numbers of neutrophils. This emphasizes the need to improve proteomics approaches to analyze ‘few-cell proteomes’ of neutrophils from different origins.

Here, we demonstrate the efficacy of an existing S-trap (micro) protocol (24) with slight modifications in protein digestion, dealing with sample amounts in the nanogram range (≤100 ng) in a highly reproducible manner. For mass spectrometric (MS) analysis, we employed the python package for DIA-PASEF methods with automated isolation design (25) (py-diAID) optimized data-independent acquisition parallel accumulation serial fragmentation (DIA-PASEF) method, which has displayed significant potential in enhancing proteome coverage with remarkable sensitivity and speed from isolated single cells (26, 27). Through this integrated workflow, we achieved a proteome coverage of over 4700 and 3400 proteins, typically ranged from 10^2^ to 10^8^ copies per cell, from 1000 circulatory neutrophils from mice and humans, respectively. Additionally, we validated the MS copy number estimation with quantitative flow cytometry. Next to direct comparisons of murine and human cells, our comprehensive proteomic analysis provides insights into the distinct molecular adaptations of neutrophils to inflammatory contexts (stroke and transient oral inflammation) compared to their circulating counterparts. Overall, this study offers a highly reproducible and sensitive approach for in-depth analyses of the neutrophil proteome with low sample quantity.

## Experimental Procedures

### Sample Cohort

C57BL/6JHsd WT male mice, aged 8 to 10 weeks acquired from Envigo were included in this study for method optimization and stroke research. All experiments were conducted with the ethical approval of the State Office of Nature, Environment and Consumer Protection North Rhine-Westphalia (G1719/19) following the EU Directive 2010/63/EU.

Five healthy male individuals within the age group 25 to 35 years with no known chronic disease or clinical signs of acute infection voluntarily participated in this study. Blood from these donors was collected to a 7 ml EDTA-coated blood collection tube (01.1605, Sarstedt AG) between 09:00 AM and 10:00 AM by a medical professional in University Hospital Essen, and samples were stored on ice until further processing, as recommended (28). This study is ethically approved by the Institutional Ethics Committee of the Medical Faculty of the University Duisburg-Essen (internal number 21-10184-BO). To study the transmigrated neutrophils, seven healthy individuals (25–35years) from University of Münster were included. The study is performed with the approval of Ethik-Kommission Westfalen-Lippe (2021-424-f-S). The human studies in this work abide by the Declaration of Helsinki principles.

### Isolation of Circulatory Neutrophils

Neutrophils were extracted from both human and murine blood using a magnetic negative isolation technique according to the manufacturer's instructions within 30 min after blood collection. Before this isolation, erythrolysis was carried out on mouse whole blood samples using in-house–prepared RBC lysis buffer (150 mM NH4Cl, 10 mM KHCO3, 0.1 mM EDTA, pH 7.2) followed by Neutrophil Isolation Kit (130-097-658, Miltenyi Biotec) on ice. Human whole blood was processed with the MACSxpress Whole Blood Neutrophil Isolation Kit (130-104-434, Miltenyi Biotec) and afterward with MACSxpress Erythrocyte Depletion Kit (130-098-196, Miltenyi Biotec) at RT. Following isolation, the cells were washed twice with PBS and cell counts were determined using a cell counter. Subsequently, 100,000 and 1000 neutrophils were enumerated and stored at −80 °C until further utilization or used directly in the case of quantitative flow cytometry.

### Sample Preparation for Proteomics Analysis

The neutrophil pellet was subjected to lysis using a 1% SDS buffer and then sonicated for 10 min utilizing a Bioruptor (Diagenode). The SDS concentration of the lysate was increased to 4% and the lysate was incubated at 95 °C for 10 min. The protein content was quantified using both a bicinchoninic acid assay and amino acid analysis. The entire cell lysate was reduced with 10 mM DTT, alkylated with 20 mM iodoacetamide, and digested using S-Trap micro spin columns (Protifi) with slight modifications to the manufacturer's protocol. Briefly, the proteins were acidified with phosphoric acid (89%) to achieve a final concentration of 6% (final pH of the solution <1). First, S-trap microcolumns were activated with the binding buffer (composed of 90% methanol and 10% tetraethylammonium bromide at pH 7.6) followed by immobilizing the acidified proteins on the columns. Contaminants were removed from the columns by washing three times with an S-trap binding buffer. To digest proteins efficiently, sequencing grade trypsin was used at a 1:2 ratio, instead of adding 1 μg as suggested by the vendor to prevent interference with further MS analysis. Additionally, we used 10 mM calcium chloride to facilitate the trypsin activity at high temperatures (29). Finally, the digestion buffer, consisting of 10 mM CaCl_2_, 50 mM ammonium bicarbonate (ABC), and trypsin was then added on top of the column and incubated for 2 h at 47 °C within a wet chamber. Peptides were eluted with 50 mM ABC followed by 0.2% formic acid, and 50% acetonitrile (ACN). Subsequently, they were vacuum-dried and reconstituted in a 0.1% TFA solution for subsequent proteomic analysis.

### Spectral Library Generation

To generate an organism-specific neutrophil spectral library, peptides were pooled from the bulk digest of five individuals. Finally, pooled peptides equivalent to 250,000 cells for each organism were subjected to high-pH fractionation (pH = 8) using the UltiMate 3000 Nano liquid chromatography (LC) system (Dionex). To fractionate the peptides, separation was carried out at pH 8 using a 15 cm C18 reverse-phase column (YMC), with a 105-min linear gradient ranging from 7% to 45% solvent B (composed of 10 mM ammonium formate and 84% ACN). A total of 16 concatenated fractions were collected, followed by vacuum drying and reconstitution in a 0.1% TFA solution. Before LC-MS/MS analysis, all fractions were augmented with iRT peptides (Biognosys, SKU:Ki-3002-1) as internal reference standards. Approximately one-third of the fractions were first concentrated on a C-18 trap column (100 μm diameter x 2 cm length, Acclaim Pepemap100) before separation on a 25 cm long and 75 μm I.D. C-18 reversed-phase Aurora column equipped with an integrated emitter (Ion Opticks) at 400 nl min^−1^ flow rate. Separation was achieved using a linear gradient of 3% to 35% solvent B (composed of 84% ACN and 0.1% formic acid) over 90 min, with the column oven set to 50 °C. Mass spectrometry analysis was performed on a timsTOF HT instrument (Bruker Daltonics) in a data-dependent acquisition PASEF mode. Each acquisition cycle included one MS1 scan followed by 10 PASEF MS/MS scans within 100 to 1700 m/z range. Ion accumulation and ramp times were set at 100 ms. All ions were analyzed within the ion mobility (1/K0) range from 1.6 Vs cm−2 to 0.6 Vs cm−2 and within the MS scan range. The collision energy was adjusted based on ion mobility, starting at 59 eV at 1/K0 = 1.6 VS cm−2 and decreasing to 20 eV at 1/K0 = 0.6 VS cm−2. Dynamic exclusion was set at 40 s. To generate the spectral library, all raw files corresponding to each fraction were analyzed on Spectronaut v.18 (Biognosys) using the built-in search engine Pulsar with Biognosys (BGS) default settings. The databases used for protein identification from mouse and human neutrophil peptide fractions are UniProt mouse database (UP000000589, 17,174 sequences, downloaded on August 29, 2023) and UniProt human database, respectively (UP000005640, 20,404 sequences, downloaded on January 30, 2023). To identify the peptide, Trypsin/P was set as enzyme with a specific digest type, and peptide lengths between 7 to 52 were considered. Missed cleavages were allowed up to 2. Carbamidomethylation at cysteine (+57.021 Da) was set as a fixed modification, whereas acetylation of the protein N terminus (+42.011 Da) and oxidation at methionine (+15.995 Da) were set for variable modifications. Following BGS default settings, both precursor (MS1) and fragment (MS2) tolerances were set as dynamic with a correction factor of 1. An intensity-based fragment ion selection strategy was implied. Protein, peptide, and peptide spectrum matches were identified with a 1% false discovery rate (FDR).

### DIA-PASEF Analysis

Based on the spectral library, DIA PASEF method was optimized using the py-diAID package, with 2 ion mobility (1/K0) windows covering a range from 0.6 to 1.6 Vs cm^−^^2^ and a precursor isolation window from 300 to 1200 m/z. This optimization resulted in 15 scan events with variable mobility and mass window, each with an accumulation time of 130 ms and ramp time, leading to a cycle time of ∼2.1s. This scheme included eight PASEF ramps and one MS1 survey. Other parameters remained consistent with those used in the data-dependent acquisition PASEF method. From the bulk digest (100,000 cells), we injected a maximum of 400 ng (∼4000 cells) as standard input. One thousand cell equivalent from the bulk digest and the entire digest of 1000 cells were analyzed on timsTOF HT with an optimized DIA PASEF scheme for 80 min. Human-specific neutrophil library comprising 5140 proteins, 55,254 peptides, and 79,666 precursors and mouse neutrophil library with 6106 proteins, 67,573 peptides, and 90,213 precursors was further used to analyze the DIA PASEF raw files on Spectronaut v.18 (Biognosys) using BGS default settings. The extraction window for ion mobility and retention time, MS1 and MS2 tolerance was set as dynamic. Calibration of all runs was done using iRT with high accuracy. Precursors and proteins were identified with 1% FDR. For peptide quantification, three precursors per peptide and the area under the MS2 signal were used. Gene Ontology (GO) analysis was performed with DAVID webserver (DAVID knowledgebase v2023q4) (30). For GO analysis, we have curated the GO terms, that is, biological processes and cellular components using QuickGO (31). The descendant terms that have a direct relationship with the parent term were considered as single GO term and their mapped genes were counted together for the parent term. For the curation, the terms that passed a *p*-value <0.05 adjusted with Benjamini–Hochberg method (32) were taken forward and ranked based on their gene count.

### Copy Number Validation with Quantitative Flow Cytometry

We selected three proteins CD11b, CXCR2, CD82 from human and CD11b, CXCR2, Ly-6G from mouse proteome data for copy number validation with flow cytometry based on their abundance and antibody availability. Mouse or human-purified neutrophils were distributed at 50,000 cells per well into a 96-well V-bottom plate (Greiner bio-one, Cat. No. 651201). FcR-mediated binding of antibodies was blocked by the incubation of cells for 10 min at room temperature with either 10% (v/v) rat serum (Thermo Fisher Scientific, Cat. No. 10710C), 1% (v/v) TruStain FcX Plus (BioLegend, Cat. No. 156604) in D-PBS (in case of mouse neutrophils), or 5% (v/v) Human TruStain FcX (BioLegend, Cat. No. 422302) in BD Stain Buffer (BD Biosciences, Cat. No. 554657; in case of human neutrophils). Subsequently, a 7-point antibody dilution ranging from 1000 ng to 15.63 ng per test was prepared in BD Stain Buffer, and cells were incubated for 30 min at 4 °C with the prepared antibody dilutions. The following antibodies were used: anti-mouse/human CD11b-PE (Cat. No. 101208), anti-mouse Ly-6G-PE (Cat. No. 127608), anti-human CD182 (CXCR2)-PE (Cat. No. 320706), anti-mouse CD182 (CXCR2)-PE (Cat. No. 149303), anti-human CD82-PE (Cat. No. 342104). All antibodies were purchased from BioLegend. Cells were then washed thrice in BD Stain Buffer and data was recorded on a Cytek Aurora (5L, Cytek Biosciences) instrument, using the pre-configured “CytekAssaySettings” gain settings. Singlet neutrophil events were gated *via* FSC-H *versus* FSC-A scatter signal and the median fluorescence intensity (MdnFI) of the PE peak channel YG1-A was exported for each antibody dilution step. To identify the optimal concentration for surface molecule saturation of each antibody used, MdnFI values were imported into GraphPad Prism (GraphPad Software) and antibody amount per titration step was plotted against MdnFI. Consequently, a saturation curve was fitted using a custom nonlinear regression model with the following formula as described in a previous study (33).

$$\text{y}=\text{y}0+{\text{A}}^{*}\text{e}{{}^{}}^{\left({\text{R0}}^{\text{∗}}\text{x}\right)}$$

Initial values were set to 1∗YMAX for y0 and to −1 (to be fit) for A and R0, constraining both A and R0 to values less than 0. For each antibody titration, the resulting values for y0, A, and R0 were transferred to a Microsoft Excel (Microsoft Corporation) spreadsheet and y0 was recalculated to correspond to 99.95% of the initial value that we determined as an optimal balance between precision and antibody consumption. Using this recalculated y0 as well as A and R0, Excel’s Goal Seek function was used to solve the above equation for x, resulting in the optimal antibody amount necessary to fully saturate all surface molecules. Fifty thousand neutrophils per well of the same batch were then again transferred to a 96-well, V-bottom plate and FcR-blocked as described above. The optimal antibody amount calculated as described above was then used to stain surface target molecules for 30 min at 4 °C. Afterwards, cells were washed thrice with BD Stain Buffer and fixed in 1% (w/v) paraformaldehyde (Merck, Cat. No. P6148-500G) in BD Stain Buffer for 20 min at room temperature. Permeabilization was performed for 15 min at room temperature using 0.2% (v/v) Tween-20 (Carl Roth, Cat. No. 9127.1) in BD Stain Buffer. Cells were then again blocked using either 10% (v/v) rat serum, 1% (v/v) TruStain FcX Plus in D-PBS (in case of mouse neutrophils), or 5% (v/v) Human TruStain FcX in BD Stain Buffer (in case of human neutrophils), and another 7-point titration was performed using antibody dilutions ranging from 1000 ng to 15.63 ng per test in BD Stain Buffer for 1 h at room temperature to account for the intracellular portion of the target protein. Cells were again washed thrice in BD Stain Buffer and data was recorded on a Cytek Aurora 5L flow cytometer, using the same analysis strategy employed as described for the surface staining. For a later conversion of MdnFI values to absolute counts, BD QuantiBrite Beads (BD Biosciences, Cat. No. 34095) with a known coupling rate of beads to PE molecules were recorded on the Aurora according to the manufacturer’s instructions using the same gain settings used for the cell acquisition. The MdnFI values were log_10_-converted, and a calibration curve was generated in Microsoft Excel, thereby extracting the trendline equation. For each target protein, a final MdnFI value was then extrapolated as follows: first, the ratio of MdnFI signal of the surface stain without cell fixation *versus* the MdnFI signal of surface-stained, fixed, and permeabilized cells was calculated to account for a loss of molecules due to the permeabilization procedure. Next, the MdnFI value for the combined surface staining and intracellular staining at the optimal concentration was calculated *via* saturation curve fitting and identification of y0 as described above. The resulting y0 was then upscaled by the loss factor ratio and finally log_10_-converted. Using the trendline equation established on the QuantiBrite beads, the log_10_-converted MdnFI value was translated into log_10_ PE molecule numbers and then into absolute molecule counts by reversing the log_10_ function and multiplying the result with the antibody lot-specific coupling rate (“F:P ratio”).

### Comparative Proteomic Analysis Between Neutrophils Isolated from Brain and Circulation Post Brain Injury

Ischemic brain injury was induced by a 60 min transient middle cerebral artery occlusion in C57BL/6JHsd male mice (aged 8 − 10 weeks) anesthetized with 1% isoflurane in 100% oxygen. Mice were injected with the analgesic buprenorphine (0.1 mg/kg body weight, s.c.) 30 min before the surgery. An eye ointment (Bepanthen) was applied to avoid any harm to mouse eyes during the surgical procedures. A small incision was made between the ear and the eye to expose the temporal bone, and a laser Doppler flow probe was attached to the skull above the core of the middle cerebral artery territory. Mice were then placed in a supine position on a feedback-controlled heat pad, and the midline neck region was exposed with a small incision. The common carotid artery and left external carotid arteries were identified and ligated. A 2 mm silicon-coated filament (Cat. 702234PK5Re; Doccol) was inserted into the internal carotid artery to occlude the middle cerebral artery. Brain ischemia was validated by a stable reduction of blood flow to ≤20% of baseline that was observed on the laser Doppler flow device. After 60 min of occlusion, the filament was removed for the reestablishment of blood flow. Mice were then injected with the anti-inflammatory drug carprofen (4 mg/kg body weight, s.c.), which was administered daily, and wounds were carefully sutured, and mice returned to their cages with free access to food and water. The exclusion criteria for experimental mice were as follows: inadequate ischemia (reduction of blood flow does not reach ≤20% of baseline), excessive weight loss (>20% of baseline), and spontaneous animal death. Twenty four hours after stroke surgery, mice were deeply anesthetized with ketamine/xylazine (100 mg/kg/10 mg/kg, i.p.) and blood was collected *via* cardiac puncture and added to EDTA-containing tubes. Mice were then perfused with PBS and brains were removed and directly transferred to ice-cold HBSS, containing 15 mM Hepes buffer and 5% glucose. The ipsilateral hemisphere was isolated and chopped down into small pieces, centrifuged at 350*g* for 3 min, and transferred into digestion mix with Dulbecco's Modified Eagle Medium, 10% fetal calf serum, penicillin-streptomycin, 10 μg/ml DNase-I, and 0.15 U/ml liberase. After incubation at 37 °C for 10 min at 80 rpm on a shaker, the suspensions were resuspended *via* a 20-gauge needle and passed through a 40 μm cell strainer. Afterwards, suspensions were centrifuged at 350*g* and resuspended in 40% Percoll solution. This solution was overlaid on 70% Percoll solution and centrifuged for 30 min at 2100 rpm, followed by lipid layer removal and cell interphase isolation. The isolated cell interphase was washed with PBS and further processed with mouse neutrophil isolation kit (MACS) according to the manufacturer’s instructions. One thousand isolated neutrophils derived from both brain and blood were taken forward for proteomic analysis. Sample preparation for proteomic analysis and subsequent LC-MS/MS analysis were conducted using the above-mentioned optimized method. In the analysis of MS raw data, precursors that met the Q-value threshold (≤0.01) were considered for protein group quantification. The label-free quantification of proteins was executed using the 'MaxLFQ' algorithm, allowing for the inclusion of three peptides per protein in the quantification process. Data normalization was achieved through a local normalization strategy based on retention time–dependent logistic regression models across the run. For subsequent statistical analysis, the log2-transformed intensity values at both the MS1 and MS2 levels were considered for paired t-tests. Candidates exhibiting a log2 fold change (brain/blood neutrophils) ≥ ±1 and a *p*-value <0.05 were considered statistically significant.

### Proteome Analysis of Neutrophils Isolated from Human Mouthwash upon Tabasco Treatment

To isolate transmigrated neutrophils from the oral cavity, seven healthy human individuals (four males and three females) washed their mouths with a Tabasco solution (10% in saline) for 30 s. After 2 h without drinking or eating, the oral cavity was washed three times with saline, and the mouthwash (oral lavage) was collected. Cells from the oral cavity were filtered two times using 30 μm filters before further processing. Blood from the same donors was drawn into an EDTA tube at the same time point, and erythrocytes were lysed using RBC lysis buffer (BioLegend, 20 min on ice) before further processing. Cell suspensions were stained with anti-CD66b (Cat. No. 305103) and anti-CD16 (Cat. No. 302027) antibodies in Cell Staining Buffer (BioLegend). After the exclusion of doublets based on scatter characteristics and dead cells with DAPI (4′,6-diamidino-2-phenylindole), neutrophils were sorted as defined as CD66b+/CD16+ (blood) or CD66+ (oral cavity) using a Sony SH800 cell sorter. For flow cytometry analysis, isolated cells were stained with the following antibodies (purchased from BioLegend): anti-CD45 (Cat. No. 103127), anti-CD66b (Cat. No. 305103), anti-CD15 (Cat. No. 702057), anti-CD11b (Cat. No. 301327), and anti-CD62L (Cat. No. 304805). Data acquisition was performed on a Cytek Aurora (5L, Cytek Biosciences) and data were analyzed using FlowJo v10.10.0 (BD Bioscience). To analyze the neutrophil proteome from 1000 fluorescence-activated cell sorting cells, sample processing, LC-MS analysis, and raw data processing were conducted using the above-mentioned optimized method. Candidates passing the paired *t* test (*p* value<0.05) with log2 fold change (lavage/blood neutrophils) ≥ ±1 were considered statistically significant.

### Experimental Design and Statistical Rationale

This study generated a total of 62 MS data files which have been uploaded in PRIDE. It includes 10 samples (five *Mus musculus* and five *Homo sapiens*) each with 10^5^ circulating neutrophils for benchmarking the method for the 1000 neutrophil proteome analysis. The protein digests obtained from the same samples were pooled and fractionated at high pH to generate organism-specific spectral libraries. Using the aforementioned DIA PASEF scheme (uploaded in PRIDE), 1000 cells were measured in five biological replicates. Further, iRT peptides were spiked in for the normalization of retention time across the runs. Additionally, we measured the samples from the lowest to the highest amount of sample input to avoid potential carryover effects from high input. To employ this method in the inflammation study, three mice were included in the stroke sample (ipsilateral hemisphere of ischemic brain, n = 3 and blood, n = 3). This approach also supported the 3R guidelines for animal experimentation (34) as it allows to strongly reduce the number of required experimental animals since all individuals in a study can be analyzed separately, which provides statistically sound data with much smaller group sizes than previously possible. For the human transmigration study, seven individuals (Lavage, n = 7 and blood, n = 7) were considered. Statistical significance for differences in protein identification was determined by student’s *t* test in GraphPad Prism v.9. Data presented as mean ± SD, where ∗∗*p* < 0.01, ∗∗∗*p* < 0.001, ∗∗∗∗*p* < 0.0001. For paired samples, the paired *t* test was used. Pearson correlations, heat maps, and volcano plots for differential expression analyses were made in Science and Research online (SR) plot.

## Results

### Species-Specific Proteome Libraries for Human and Mouse Neutrophils

**Fig. 1:**
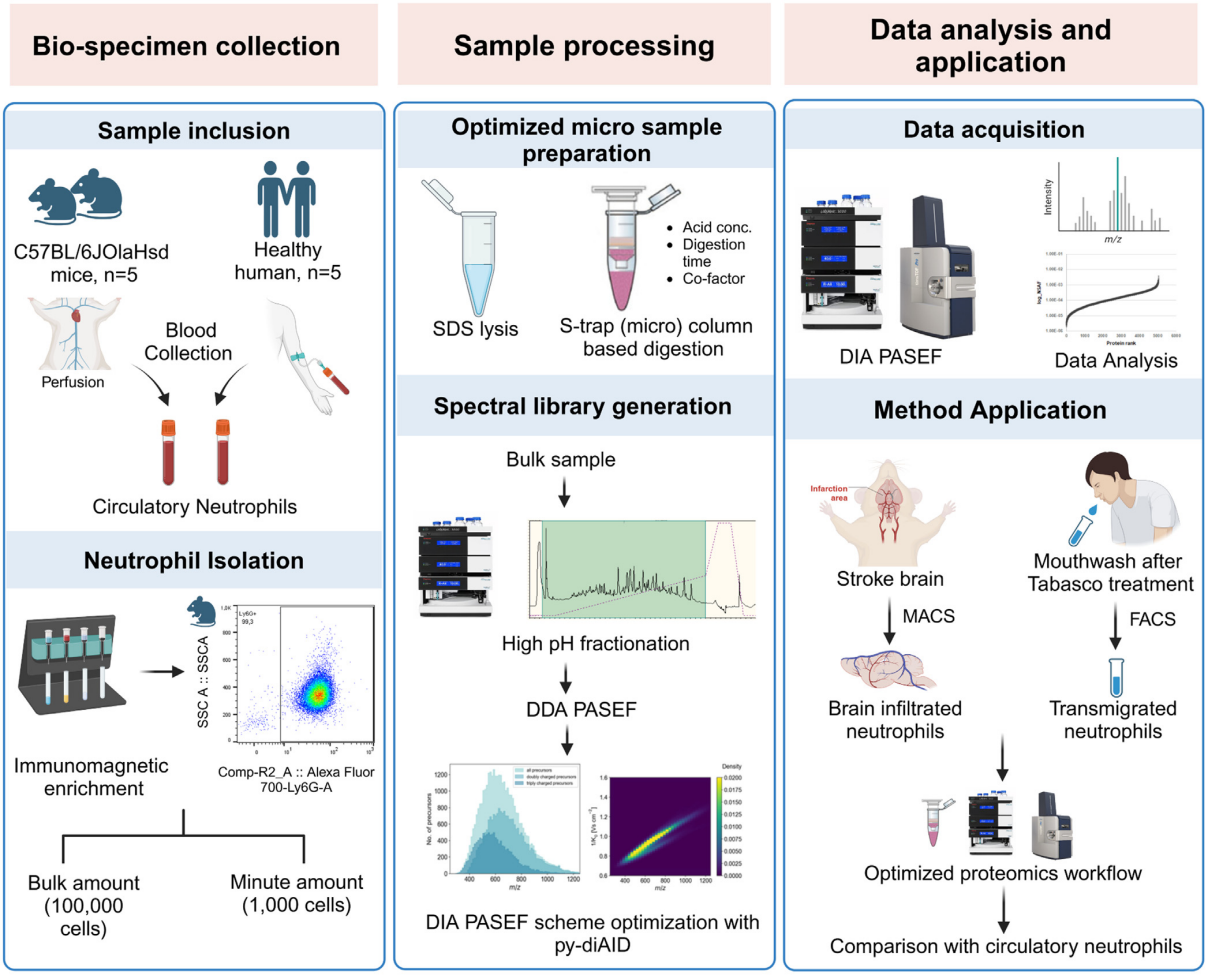
**Workflow for analyzing the proteome of murine and human neutrophils isolated from blood and sterile inflammatory sites.** The sample cohort included healthy human and naive mouse blood neutrophils with five biological replicates that were utilized for the establishment of a 1000-cell proteome method. Neutrophils isolated from mouse ischemic brain and the inflamed human oral cavity were used for the validation of established technologies.

Multiple technologies exist to isolate high-purity neutrophils from blood. In our study, we achieved a high level of purity for both (∼97–98% of CD45+ cells) human (CD66b+) and mouse (Ly-6G+) neutrophils through the application of magnetic bead-based negative isolation as described (35). While more than a million pure neutrophils were obtained from 1 ml of human blood, isolation from mouse blood reproducibly yielded 0.1 million (10^5^) isolated neutrophils using MACS-based negative isolation. Therefore, we established this as the standard input for our proteome study (Fig. 1).A spectral library was constructed for the human resting circulatory neutrophil proteome, comprising 16 peptide fractions, and encompassing a total of 5140 proteins, 55,254 peptides, and 79,666 precursors, predominantly doubly and triply charged (supplemental Fig. S1*A*). Employing a Bayesian algorithm-based optimal scheduling scheme within the py-diAID interface, we achieved ∼99% protein coverage from the spectral library in its DIA-PASEF scheme (supplemental Fig. S1*B*). To assess the comparability of our library with the data previously reported by Grabowski *et al*. (36), we downloaded raw files corresponding to healthy human neutrophils and re-analyzed them with our database and search settings. Our analysis covered approximately 73% of the proteins from their library and 444 (7%) additional proteins despite having 10 times less starting material (supplemental Fig. S1*E* and supplemental Table S1). Importantly, both libraries successfully identified proteins involved in neutrophil effector functions and cellular processes, indicating the suitability of our approach in understanding key aspects of neutrophil function with low sample quantity. Our spectral library for murine circulatory neutrophils comprised 6106 proteins, 67,573 peptides, and 90,213 precursors, which was ∼4% more than a recently published combined spectral library for mouse bone marrow, blood, and peritoneal neutrophils (37). Compared to the human neutrophil library, the mouse library showed a substantially higher number (∼15%) in precursor identifications and identified proteins, despite the same amount of source material and fractions. This was irrespective of the fact that human neutrophils are ∼1.8-fold larger than murine cells and consequently contain 1.8-fold more protein per cell (supplemental Fig. S1*F* and supplemental Table S1). The distribution of charged precursors across the mass-to-charge ratio (m/z) plane closely resembled that of the human library. The optimized DIA-PASEF scheme for mouse neutrophils covered 99% of the proteins in the spectral library (supplemental Fig. S1, *C* and *D*).

### Optimized Method for Protein Profiling of 1000 Circulatory Neutrophils and Identification of Key Pathways

**Fig. 2:**
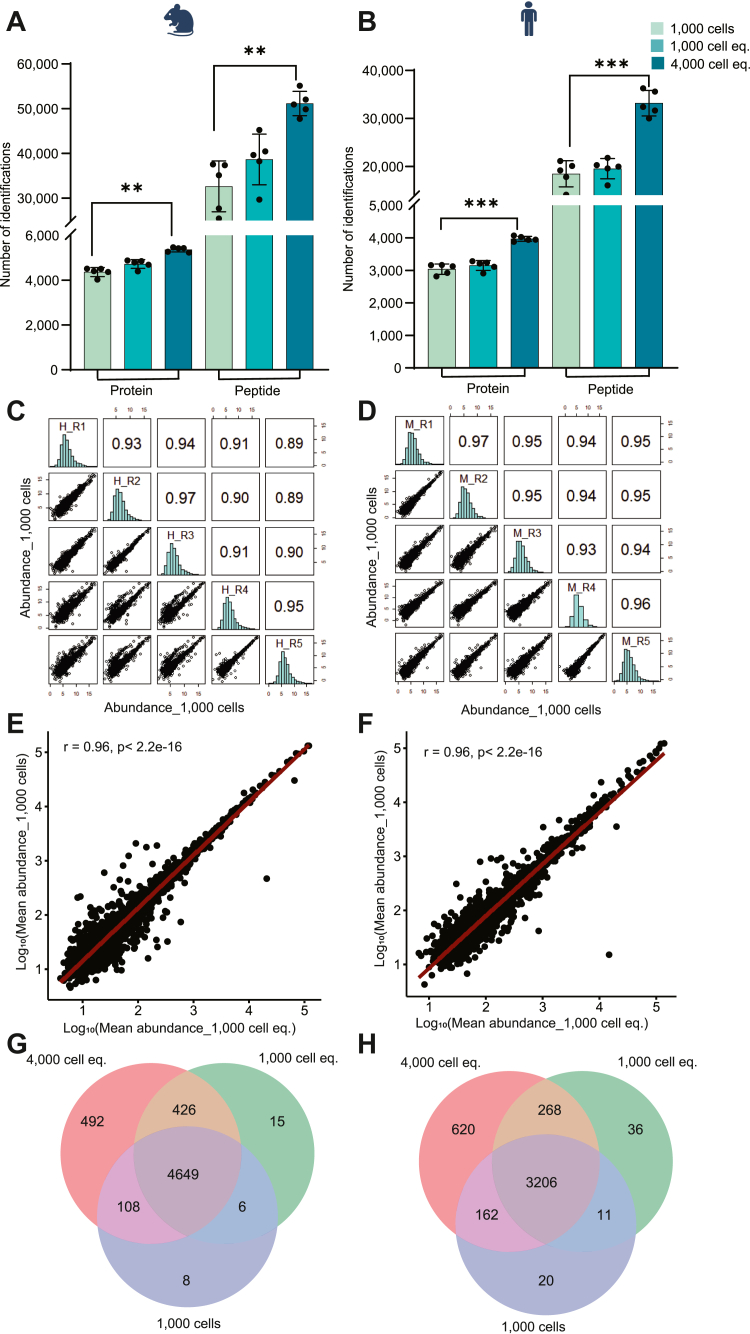
**Protein profiling of 1000 neutrophils isolated from human and mouse blood (n = 5)**. *A* and *B*, comparison of proteins and peptides identified from a 1000 cell digest, 1000 cell equivalent (eq.), and 4000 cell eq. 1000 and 4000 cell equivalents were derived from standard digests (100,000 cells). Data are represented as mean ± SD. Statistical significance was determined by paired *t* test, where ∗∗*p* < 0.01, ∗∗∗*p* < 0.001. *C* and *D*, the correlation among biological replicates of 1000 cells based on protein quantity. *E* and *F*, proteome correlation between 1000 cells and 1000 cell eq. This includes the proteins present in both conditions. *G* and *H*, Venn diagram showing the overlap of proteins identified from different conditions.

With the spectral library as maximum benchmark, we next analyzed the proteome of 1000 neutrophils from mouse or human blood. To account for potential unspecific sample loss, we also analyzed 1000 or 4000 cell equivalents (100–400 ng for human neutrophils) obtained from a standard input (100,000 cells). We set 4000 cell equivalents as the maximum injection amount in our analysis as beyond that we observed a slight column saturation. On average, more than 4300 mouse and 3000 human proteins were identified from 1000 neutrophils (Fig. 2, *A* and *B*) with a strong correlation (Pearson correlation coefficient >0.9) across five biological replicates (Fig. 2, *C* and *D*). Prior to further analysis, we excluded the proteins that are typically considered universal contaminants in proteomic studies arising from reagents or sample handling (38) (supplemental Table S2). Additionally, we detected immunoglobulins (*e.g.*, lambda and kappa chain variants) and hemoglobin subunits for both organisms which are likely derived from the pre-isolation erythrocyte lysis, antibodies used during the isolation and carry-overs from blood plasma. Hence, the analysis of 1000 cells yields 78.5% of the bulk proteome (*i.e.* the spectral library) in murine and 66.5% in human neutrophils.

The proteome coverage in 1000 isolated cells was lower (4% in human and 8% in mouse) than that in 1000 cell equivalents from the standard digest, which can be accounted to sample loss/absorption during preparation (Fig. 2, *A* and *B*). Nevertheless, the average protein abundance in 1000 cells and 1000 cell equivalents correlating well (r = 0.96) confirms the robustness of the method (Fig. 2, *E* and *F*). Further, we analyzed 4000 cell equivalents from the standard digest which yielded on average 5365 identified proteins for mouse and 3972 for human neutrophils from five biological replicates (Fig. 2, *A* and *B*). Similar to low input proteome, 4000 cell analyses showed reasonable correlation (Pearson correlation >0.9) in expression across replicates(supplemental Fig. S2, *A* and *B*). This 23 to 30% increased identification in the 4000 cell equivalents (eq.) compared to 1000 cell eq. or 1000 isolated cells highlights the challenges of low input measurements. This is also reflected in data completeness where ∼70% of the total identifications were found to be consistently present in all replicates of 1000 cells but that number was 80% in 4000 cells eq. (supplemental Fig. S2, *C* and *D*). Despite the loss in 1000-cell analyses, our method still achieved a remarkable coverage, capturing ∼80% of the standard input proteome in both human and mouse neutrophils (Fig. 2, *G* and *H*). This included key proteins involved in neutrophil activation (myeloperoxidase (Mpo), CD63, lipocalin-2 (Lcn2), neutrophil elastase (Elane), carcinoembryonic antigen-related cell adhesion molecule (CEACAM8/CD66b for human), integrin alpha M (Itgam/CD11b), L-selectin (CD62-L)), maturation (FcγR1-4, CD101, CD14, CXCR2, CD47), migration (Itgb2, CD177), chemokines like CXCL2, CXCL6, CXCL7, and other proteins associated with cellular function and metabolism. In contrast, low abundant proteins related to transcription, mRNA processing, and chromatin organization were not detectable with a low input of cells (supplemental Table S2).

**Fig. 3:**
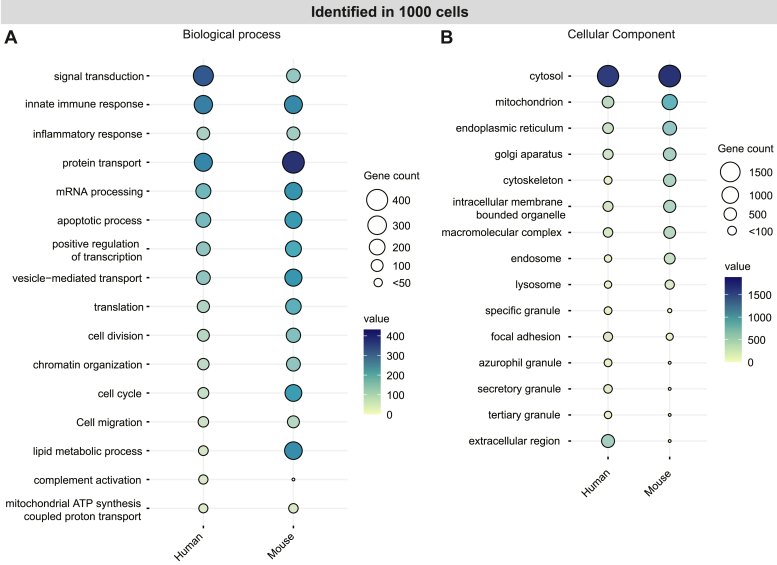
**Gene Ontology analysis of neutrophil proteomes.***A*, biological Processes and (*B*) cellular components that passed the Benjamini–Hochberg (32) adjusted *p*-value cut-off (<0.05) were ranked by gene count. The top pathways in each or both organisms are shown as a function of gene count.

The GO analysis of 1000-cell proteomes exhibited a comparable presence of biological processes like ‘innate immune response,’ ‘inflammatory response,’ and ‘cell migration’ in both species. However, the enrichment pattern diverged in these species for the processes ‘signal transduction,’ ‘protein transport,’ ‘mRNA processing,’ ‘cell cycle,’ and ‘complement activation pathway’ (Fig. 3*A*). Our findings align well with the known differences between human and mouse neutrophil granule contents (39). In human neutrophils, we identified proteins belonging to both lumen and membrane of the ‘azurophil granule,’ ‘tertiary granule,’ ‘specific granule,’ and ‘secretory granule.’ In mouse neutrophils, proteins mostly belonging to ‘specific granule’ and ‘tertiary granule’ were detectable. Additionally, proteins belonging to ‘mitochondrion,’ ‘endoplasmic reticulum,’ or ‘cytoskeleton’ were found more in mouse cells (Fig. 3*B*). Further, reactome-based pathway analyses demonstrated that the approach achieves a good coverage of proteins mapped to the ‘neutrophil degranulation’ pathway (40) in humans (87%) and mice (70%) from as few as 1000 cells. This showcases the method’s applicability in investigating neutrophil activation in both healthy and disease contexts with minimal starting material.

### Comparative Proteome of Murine and Human Neutrophils

**Fig. 4:**
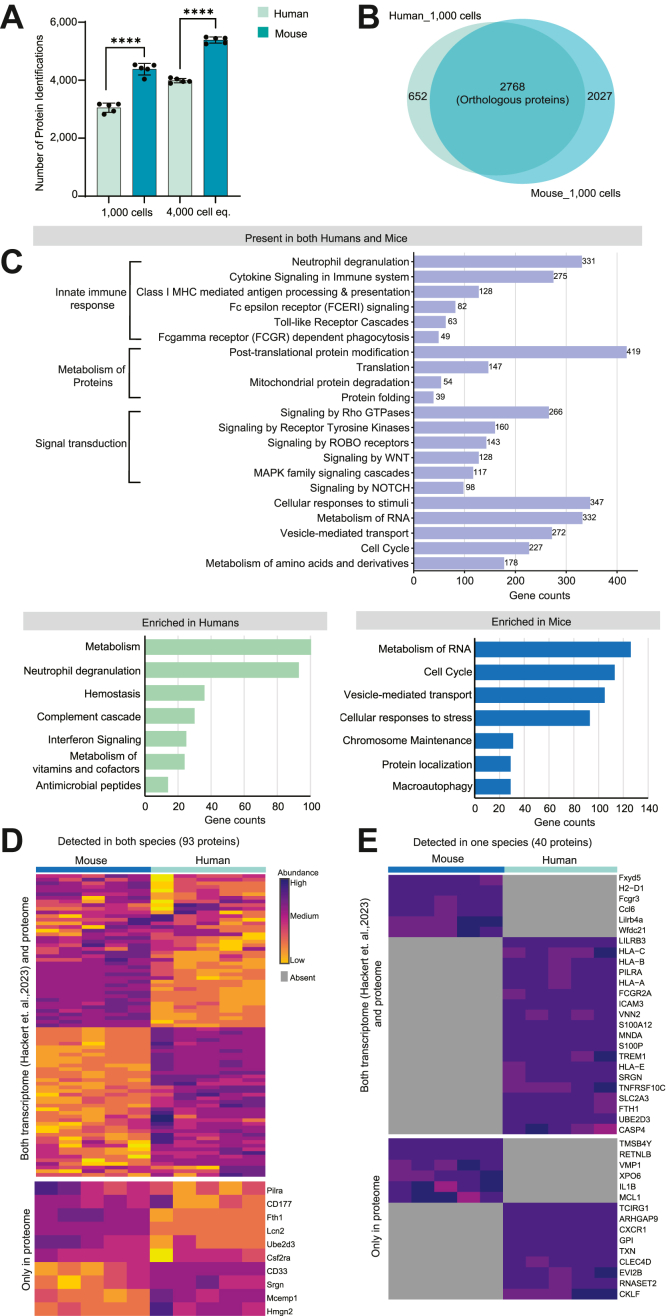
**Comparison of the protein repertoire between human and mouse neutrophils.***A*, protein and peptide identifications obtained from different cell quantities in both organisms. Data represent the mean ± s.d. of biological replicates (n = 5). ∗∗∗∗*p* < 0.0001 (two-tailed *t* test). *B*, Venn diagram illustrating shared proteins between two species, identified from the proteomics data in (*A*). *C*, reactome pathways associated with proteins identified in both organisms and uniquely detected in one organism (from *panel B*). Only pathways with Benjamini–Hochberg (32) adjusted *p*-values <0.05 are shown. *D*, expression pattern of neutrophil-lineage proteins identified in both human and mouse neutrophils compared with previously reported transcriptomics data (7). The *upper* half shows the proteins and transcripts detected in both organisms by both methods. The lower half highlights the proteins identified in both organisms but transcripts only in one organism. *E*, neutrophil-lineage proteins uniquely identified in human and mouse in accordance with transcriptomics data.

A notable finding in our present study was 20 to 25% more proteins present in neutrophils purified from mice compared to humans (also ∼15% in the spectral library). This was regardless of the number of cells analyzed (Fig. 4*A*) and despite humans having larger cell volume and protein amount than mouse neutrophils (supplemental Fig. S1*F*). This motivated us to further investigate the proteins causing this discrepancy. In our study, we found an overlap of 2768 proteins orthologous between human and mouse neutrophils when analyzing 1000 cells, accounting for ∼81% of the total identified proteins in humans, as illustrated in Figure 4*B* (supplemental Table S3). Notably, these orthologs include neutrophil lineage-associated proteins like Mpo, Elane, neutrophil cytosolic factor (Ncf1, Ncf2, Ncf), Chitinase-3-like protein 1 (Ch3l1), S100 proteins (S100A8, S100A9), matrix metalloproteinases (Mmp8, Mmp9, Mmp25), integrins (Itgb2, Itgam) receptors like Granulocyte colony-stimulating factor receptor (Csf3r), CXCR2, fMet-Leu-Phe receptors (Fpr1, Fpr2), L-selectin (CD62-L), P-selectin, ribosomal protein subunits, and the transcription factors PU.1, SP1, BTF3 besides others (supplemental Table S3).

Majority of these orthologous proteins belong to immune response pathways, for example, ‘neutrophil degranulation,’ ‘cytokine signaling in immune system,’ FCGR dependent phagocytosis, signaling cascades for example ‘signaling by Rho GTPase,’ ‘MAPK family signaling cascades,’ and other processes like cell cycle, RNA metabolism, and vesicle-mediated transport (Fig. 4*C*). This indicates a high degree of conservation for crucial pathways associated with neutrophil activity in both organisms. However, our study also identified proteins that emphasize a species-specific signature. For instance, human neutrophils are known to harbor granule proteins such as azurocidin (HBP, CAP37), bactericidal permeability-increasing protein (CAP57), defensins (Defa1, Defa3, Defa4), and surface markers including CD66b whereas lymphocyte antigen-6G (Ly-6G), neutrophil granule protein (Ngp), C-C motif chemokine 6 (CCL6), or Chitinase-like protein 3 and 4 are known to be uniquely present in mice (41, 42). These facts are also reflected in our study. In addition, we have also identified surface markers (CD66c, CD66d), caspases, complement factors, leukocyte immunoglobulin-like receptor, apolipoproteins, granzymes, and Fc receptors like FcγRII, FcγRIII uniquely in human cells. This led to a higher emphasis in human than mouse cells of pathways such as ‘neutrophil degranulation,’ ‘complement cascade,’ and ‘interferon signaling’ (Fig. 4*C*). Recently, the human and mouse neutrophil transcriptome was compared, focusing on shared neutrophil lineage genes and their gene expression pattern in inflammatory contexts (7). We could identify 133 of 182 genes mentioned in the transcriptomics dataset in our 1000 cell proteome data. By comparing these two datasets, we observed a coherent expression of those orthologs present either in both species or only in one species (Fig. 4, *D* and *E*). Interestingly, we identified 10 proteins including CD33, CD177, and Lcn2, in both species in proteome data but only in mouse transcriptomics data. However, previous studies (43, 44, 45) have already shown the presence of these proteins in human neutrophils. On the other hand, we found 13 proteins uniquely in human and 11 in mouse cells, which were identified in both species according to transcriptomics data (supplemental Table S3). These discrepancies highlight the necessity for a validation of transcriptome data with proteome analyses to get a robust information on the molecular makeup of human and mouse neutrophils.

While our study provides insight into the species-specific variations in neutrophil proteomes, the reason for the substantially increased number of expressed proteins in murine neutrophils remains elusive, especially in light of the significantly larger volume and protein mass of human than mouse neutrophils (supplemental Fig. S1*F*). Along with species-specific neutrophil lineage genes, the majority of the excess proteins seen exclusively in murine neutrophils are primarily associated with pathways such as ‘metabolism of RNA,’ ‘cell cycle,’ and ‘vesicle-mediated transport’ (Fig. 4*C*), These enrichments likely reflect known species-specific differences in developmental rates, cell cycle duration, and metabolic regulation (46, 47). Further, to verify potentially increased mitochondrial protein identifications in mice compared to humans, we overlapped our data with the database downloaded from MitoCarta (v3.0) (48). This is an inventory database including over 1000 mammalian mitochondria-associated proteins (human- 1136 proteins, mouse- 1140 proteins) with their functional annotation. Our analysis demonstrated that we systematically identified 28% more mitochondrial proteins in mouse than human neutrophils (supplemental Fig. S3*A*), although principally all proteins are present in both species. While there is ongoing debate about whether human neutrophils have minimal mitochondria (49), the increased mitochondrial proteins in mice instigate further investigations to understand the species-specific difference in mitochondrial abundance and functional complexity.

### Single Neutrophil Protein Inventories with a Broad Range of Copy Numbers

**Fig. 5:**
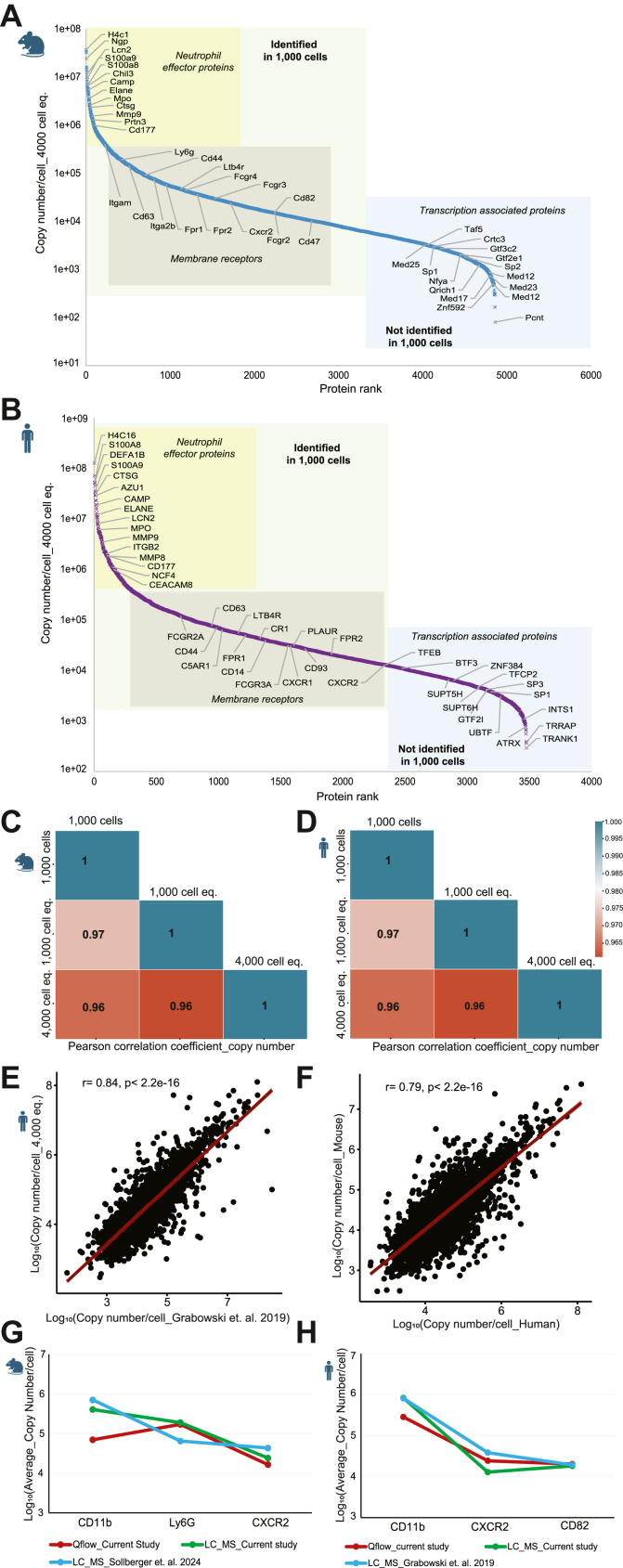
**Estimation of protein copy numbers in human and mouse neutrophils.***A* and *B*, dynamic range of protein copy numbers detected in 4000 and 1000 neutrophils. Highlighted proteins are identified in both quantities (*green* area) or not identified in 1000 cells (*blue* area). *C* and *D*, consistency of quantified protein copies across various sample quantities as indicated. Only proteins common to all three conditions were considered for the analysis. *E*, resemblance between the current study and a published repository (36) in protein copy number estimation of human neutrophils. *F*, correlation between human and mouse neutrophils based on estimated copy numbers of orthologous proteins. *G* and *H*, concordance between the quantitative flow cytometry (qFlow) and proteomics approaches in quantifying copies of surface proteins. The average of all measurements is plotted. Repository data (36, 37) were included to validate the trend.

To convert the MS signal to individual protein copies per cell, we compared two approaches Proteomic ruler (50) and the total protein approach (51). Both exhibited almost identical protein copy number estimation in human neutrophils (supplemental Fig. S2*G* and supplemental Table S4). For subsequent analysis, we employed the total protein approach to normalize copy numbers for both organisms, considering proteins consistently present in all replicates. We successfully identified proteins ranging from 10^2^ to 10^8^ copies per cell from 4000 neutrophils for both organisms (Fig. 5, *A* and *B*). An interactive searchable graph for the copy numbers is available under Supplementary file “Copy number plot_Neutrophil.html” (run in any web browser) with a detailed description in the supplementary material. Here we categorized human and mouse neutrophil proteins based on their copy numbers into high- (>5 × 10^5^), medium- (5 × 10^5^to 10^4^), and low-abundance (<10^4^) proteins, considering their detectability in 1000 cells. Histones, S100 proteins, and proteins with antimicrobial activity, for example Elane, Mpo, Ctsg, Camp, and proteases like Mmp9 and Mmp8, showed high copy numbers in neutrophils. Thereby the 150 most heavily expressed proteins together accounted for more than 80% of the total protein copies in humans and 70% of that in mice. Moreover, receptors and surface proteins such as CXCR2, FcγR, CD63, Fpr2, CD62L, and chemokines like CXCL6 and CCL6 were identified with medium-range copy numbers. Not unexpectedly, transcription-associated proteins (e.g. Smarca4, Rela), ribonucleoproteins, ubiquitin proteins, and myosin subunits were found in lower quantities (supplemental Table S4). It is worth mentioning that the transcription factor PU.1, which is crucial for granulopoiesis (52), was detected in both organisms, even with minute input. We next compared the copy number data in 4000 cells with the results obtained in 1000 cells. As anticipated, many of the low abundant proteins including the transcription factors Sp1, Sp2, ubiquitin proteins, or zinc finger proteins were not identifiable in 1000 cells, but ∼80% of all proteins in 4000 cells could also be found in 1000 cells.

Despite the challenges in detecting low-abundance proteins, the estimated protein copy number remained consistent and strongly correlated (r = 0.96) with various sample amounts (Fig. 5, *C* and *D*). This demonstrates the reliability of our measurement when using very small cell numbers for input. Additionally, our copy number estimations from 4000 cells displayed a reasonable correlation (r = 0.84 in human and r = 0.79 in mouse) with known repository data (36, 37) despite variations in sample quantity (∼10^6^ cells for repository data) and processing workflow between both approaches, thus strengthening our findings (Fig. 5*E* and supplemental Fig. S2*F*). The increased protein identifications in mouse compared to human neutrophils prompted us to compare estimated copy numbers for shared orthologous proteins. We observed a ∼1.8-fold increase in overall copy numbers in human relative to mouse cells (supplemental Fig. S3*B*), which is likely due to the larger size/protein mass of the human cells. Still, our approach also allowed a side-by-side comparison of estimated copy numbers of orthologous proteins in both species. This analysis showed a high agreement (r = 0.79) between proteins (Fig. 5*F*, (7)), hence suggesting that overall similar processes might be active in neutrophils of both species.

To verify the reliability of copy number estimation, we performed quantitative flow cytometry (qFlow) on selected protein targets for both mouse and human blood neutrophils. For mouse neutrophils, CD11b, Ly-6G, and CXCR2 were selected as prototypical targets based on the predicted abundance in the proteome data (4000 cell eq.) and antibody availability. Using titration curves (supplemental Figs. S4 and S5), we found the absolute protein copy numbers to be comparable between our two approaches. With qFlow, we found 70,054 copies of CD11b (proteomics: 408,674, 5.8-fold difference) while Ly-6G was measured at 171,919 molecules (proteomics: 190,665, 1.1-fold difference) and CXCR2 at 16,530 molecules per cell (proteomics: 24,317, 1.4-fold difference, Fig. 5*G*) in murine neutrophils.

For human neutrophils, we chose CD11b, CXCR2, and CD82 as targets for cross-verification. We obtained on average 284,704 CD11b copies per cell (proteomics: 829,809, 2.9-fold difference), indicating that human neutrophils contain considerably more CD11b than mice. CXCR2 and CD82 were detected at 24,349 and 20,200 molecules per cell, respectively (proteomics data: 12,895 copies CXCR2, 1.8-fold difference; 18,199 copies CD82, 1.1-fold difference, Fig. 5*H*). Overall, we found that the average number of target molecules was consistent between qFlow and proteomics except CD11b. As the antibody for CD11b is not well characterized, we hypothesize that the presence of glycosylation sites in the protein likely interfere with antibody binding, therefore resulting in a lower than expected copy number estimation during qFlow (53). Although the fold-differences in molecule counts increased in the CD11b between the two methods, the absolute counts remained in reasonable proximity and showed comparable trends between species. This confirms the feasibility of our method to estimate protein copies from proteome data of only 1000 cells.

### The Impact of Ischemic Brain Injury on the Proteome of Infiltrating Neutrophils

**Fig. 6:**
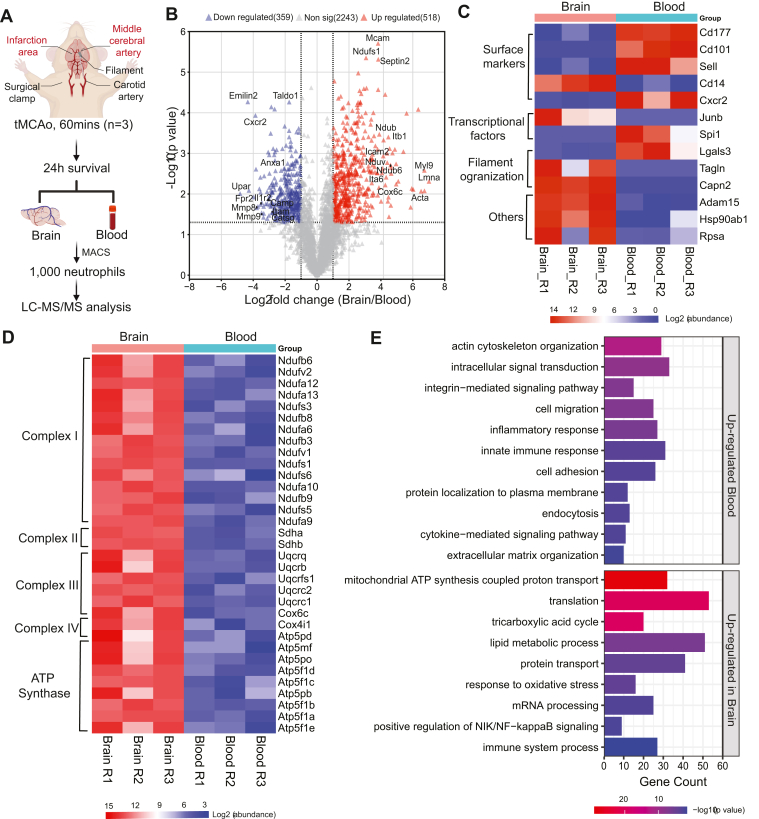
**Differential expression analysis between neutrophils isolated from murine ischemic brain and the circulation (n = 3) 24 h after tMCAO.***A*, experimental approach of stroke induction and cell isolation. *B*, volcano plot indicating proteins with significantly differential expression (Log_2_ fold change ≥ ±1, paired *t* test, *p*-value <0.05) *Red*: proteins upregulated in brain neutrophils; *blue*: proteins downregulated in brain neutrophils. *C*, expression pattern of specific proteins involved in maturation in the different neutrophil populations. *D*, overexpression of mitochondrial proteins in brain-infiltrated neutrophils 24 h after stroke. *E*, biological processes (Benjamini–Hochberg (32) adjusted *p*-value <0.05) of significantly dysregulated proteins in two populations obtained from (*B*).

Stroke is a very relevant example of sterile tissue inflammation that is massively changed by the invasion of relatively few neutrophils (14), but the molecular composition of brain-infiltrated neutrophils has so far remained elusive. Having established the reliability of our small cell-input proteome approach, we isolated 1000 neutrophils from brain and blood of the same mice after 24 h of stroke induction and compared their proteome profile (Fig. 6*A*). Our analysis yielded an average of 4255 protein identifications from each replicate and each condition which aligns with previous findings for 1000 isolated resting circulatory neutrophils. Among those, 3120 proteins were consistently quantified in all replicates and conditions with unique peptides ≥2. Subsequent statistical analyses with paired t-tests identified 877 proteins as significantly differentially expressed (log_2_ fold change of ≥ ±1 and a *p*-value of ≤0.05), with 518 being upregulated and 359 downregulated in brain-derived neutrophils (Fig. 6*B* and supplemental Table S5).

Compared to brain-infiltrated cells, blood neutrophils showed high levels of proteins involved in cell adhesion (EMILIN2 (54)), neutrophil recruitment (C5AR1) (55), brain entry (CXCR2) (56), neutrophil extracellular trap (NET) formation (TALDO1) (57), and blood-brain barrier disruption (MMP9) (58). These factors play crucial roles in acute stroke pathophysiology by facilitating immune cell brain invasion, associated blood-brain barrier damage, and NET formation that together worsen stroke outcome (59). GO analysis revealed the upregulation of migration, activation, and immune response–related proteins in blood-derived neutrophils, as characteristics of mature neutrophils (60). Consistent with these findings, our previous research demonstrated pronounced activation of circulating neutrophils in stroke-afflicted mice compared to sham controls (59). Furthermore, relative to naive mice, we detected an upregulation of proteins linked to 'neutrophil activation' and 'neutrophil chemotaxis' in circulating neutrophils following stroke onset (see supplemental Fig. S6, *A* and *B*). Collectively, these observations indicate a robust activation of circulating neutrophils within 24 h post-stroke, emphasizing their pivotal role in the acute phase of stroke pathology.

Interestingly, brain neutrophils showed signatures with a phenotype characterized by the downregulation of CD177, CD101, CD62-L, and CXCR2 while CD14 was upregulated (60, 61, 62) (Fig. 6*C*). One intriguing finding was the upregulation of mitochondrial proteins, associated with the tricarboxylic acid (TCA) cycle (*e.g.*, Idh3a, Aco2, Sdha, Idh2, Cs) in brain-infiltrated neutrophils. This suggests a metabolic flexibility of these neutrophils to survive in stress- and glucose-deprived conditions as a characteristic of CD101^-^ neutrophils (63). Furthermore, we observed a significant increase in the expression of mitochondrial electron transport chain (ETC) components in brain-infiltrated neutrophils, including subunits of complex I (Ndufv2, Ndufa12), complex II (Sdhb, Sdha), complex III (Ubqcrq, Ubqcrb), and ATP synthase subunits (Fig. 6*D*). While the stroke-induced hypoxic environment within the brain impairs aerobic respiration and ATP synthesis (64), the upregulation of ETC components might serve as an adaptive response to uphold the mitochondrial activity to sustain cellular viability under low-oxygen conditions. Our results also confirmed this *via* the GO analysis, by upregulation of ‘mitochondrial ATP synthesis coupled proton transport,’ ‘tricarboxylic acid cycle,’ and ‘respiratory electron transport’ (Fig. 6*E*).

### Molecular Changes in Human Neutrophils after Transmigration into the Inflamed Oral Cavity

**Fig. 7:**
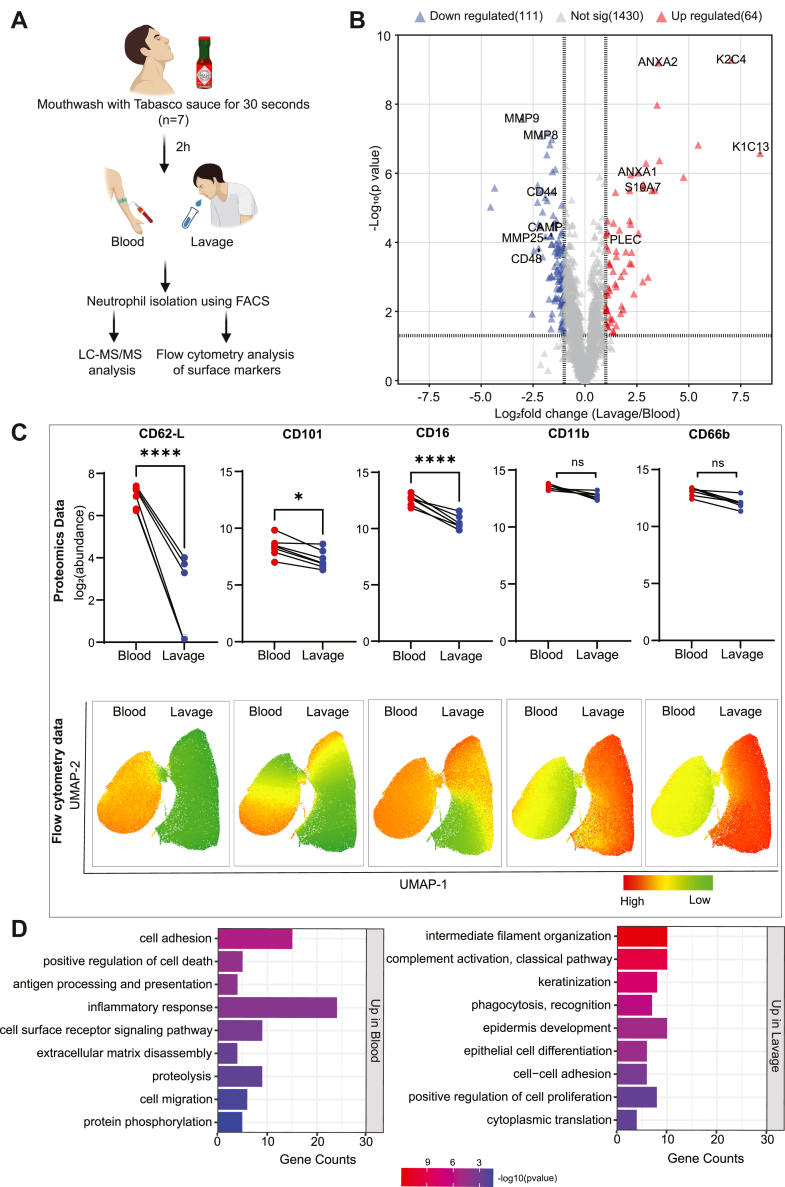
**Proteome comparison between circulatory neutrophils and neutrophils transmigrated to the oral cavity (lavage) after tabasco treatment.***A*, experimental approach of tabasco treatment and cell isolation from healthy human individuals (n = 7). *B*, volcano plot highlighting upregulated (*red*) and downregulated (*blue*) proteins in transmigrated neutrophils isolated from lavage. Statistical significance was determined by Paired *t* test with *p*-value <0.05, Log_2_ fold change ≥ ±1. *C*, paired analysis in surface markers expression in neutrophils obtained from blood and lavage samples. Analyses were made by our proteomics workflow (*top*) and spectral flow cytometry (*bottom*). Paired *t* test was used for the expression comparison (∗*p* < 0.05, ∗∗*p* < 0.01, ∗∗∗*p* < 0.001, ∗∗∗∗*p* < 0.0001). *D*, biological processes (Benjamini–Hochberg (32) adjusted *p*-value <0.05) associated with proteins upregulated either in transmigrated neutrophils or in circulatory neutrophils.

Further, we applied this approach to a local inflammation model in humans, that is, oral inflammation induced by Tabasco solution, an ethically feasible inflammation model studied in humans. While Tabasco solution is commonly used as a topical medication for burning mouth syndrome (65), mouthwashes with this solution in healthy individuals have also been utilized as a model for acute neurogenic inflammation to study human neutrophils (66). The conservation of core inflammatory responses across different scenarios in both organisms (7) supports the relevance and applicability of our findings from two different inflammatory models. Here, we evaluated the impact of transmigration on protein expression in human neutrophils from the oral cavity 2 hours after exposure to tabasco solution in comparison to blood neutrophils from the same donors (Fig. 7*A*). Our analysis of blood and oral cavity lavage-derived neutrophils (n = 7) yielded in total 3452 identified proteins, of which 1605 were identified in all replicates and all conditions with 1% FDR and were taken forward for further statistical analysis. A set of 175 proteins was found significantly differentially regulated between two groups (log_2_ fold change of ≥ ±1 and a *p*-value of 0.05). One hundred eleven proteins were upregulated in blood and sixty four in oral lavage neutrophils in response to tabasco (Fig. 7*B*). Additionally, we found 381 proteins including keratins, complement proteins, apolipoproteins, and S100 proteins that were enriched in oral lavage-neutrophils but detected in less than half or no replicates of circulatory neutrophils (supplemental Table S6).

While Frankenfield *et al*. identified keratins such as KRT4, KRT5, KRT13, and KRT14 as potential contaminants in proteomics studies (38), they are known to be expressed in the healthy human oral cavity (67). Therefore, the enrichment of keratin proteins in lavage neutrophils may also result from phagocytosis of keratin filaments by neutrophils inside the oral cavity (68). In parallel, we performed flow cytometric analyses of surface marker expression on the same preparation of neutrophils and found remarkable changes in the surface expression of markers involved in migration and activation. We could reproduce this regulation using our proteomics analysis pipeline for proteins like CD62L, CD101, and CD16 (Fig. 7*C*). In contrast, CD11b and CD66b did not show differences using proteomics whereas a changed surface expression was observed using flow cytometry suggesting a potential relocation of these proteins from their intracellular pool to the surface (69). An enrichment analysis of oral cavity and blood neutrophils revealed that cell adhesion, migration, and inflammation were elevated in the blood while complement activation, phagocytosis, and tissue homeostasis were upregulated in oral cavity neutrophils, indicating a functional shift after transmigration (Fig. 7*D*).

## Discussion

Proteomics of neutrophils illuminates an individual’s immune response to external stimuli, infections, and pathophysiological conditions. Although some studies have explored the neutrophil proteome using traditional two dimensional gel electrophoresis and advanced DIA-based proteomics, they often focused solely on granule proteins or required large quantities of neutrophils (37, 70, 71). Our analysis of just 1000 isolated neutrophils opens up new opportunities to investigate localized immune responses within individual inflammatory sites without the need for pooling, thus offering a valuable tool for future research.

In low-input or single-cell proteome analysis, surfactants like Dodecyl-beta-D-maltoside are widely used due to their MS compatibility and ability to solubilize membrane proteins, yet, the role of SDS in protein solubilization is incomparable (72, 73). However, SDS removal from samples prior to MS analysis poses significant risks of losing material. The S-trap micro column efficiently removes most SDS from lysates before digestion (24). We here show the effectiveness of S-traps as an alternative to an existing minimal input protocol (74) with better proteome coverage and without the need for chemical labeling. Strategically coupling S-traps with optimized digestion conditions enhanced the efficiency of sample processing resulting in highly consistent biological replicates as demonstrated by comparable identifications between 1000 isolated cells and 1000 cell equivalents from standard digests (100,000 cells). Our study benchmarks the potential of the S-trap micro columns in dealing wide range of sample amount (ng to μg) with high reproducibility. Furthermore, py-diAID–optimized DIA-PASEF mass spectrometry schemes allowed the reproducible identification high protein numbers from 1000 neutrophils with a wide range of copy numbers. Despite sample loss during preparation and the low input of material, we still saw a strong correlation in abundance or copy numbers with much higher sample inputs which supports the reliability of our quantification.

The advancement of proteomics has revolutionized the molecular understanding of disease biology (75, 76), yet clinical research still heavily relies on antibody-based validation. Flow cytometric analyses of cell populations are widely used in drug testing, biomarker discovery, and clinical trials (77). Hence it is important that we could confirm absolute protein numbers from our proteomic analyses with data obtained from qFlow for low- to medium-abundance proteins reasonably well. For our high-abundance target CD11b, however, we observed a higher discrepancy for both human and mouse data. As the antibody for CD11b is not well characterized, we hypothesize that the presence of glycosylation sites in the protein likely interfere with antibody binding, therefore resulting in a lower than expected copy number estimation during qFlow (53). Beyond the CD11b data, the very good concordance for copy number estimation data between both methods nonetheless strengthens our proteomic approach in determining protein copies from as few as 1000 cells. The searchable database provided could thus serve as a well-curated resource for the neutrophil community.

A particularly noteworthy aspect of our investigation is the inclusion of both human and mouse neutrophils, recognizing the species-specific differences in the proteomic landscape of these immune cells. While murine cells may exhibit phenotypical similarities to human neutrophils, the differences in their life span, maturity, surface markers, signaling pathways, and the repertoire of secretable molecules limit direct extrapolations of findings from mouse to human neutrophils (42, 78, 79). The transcriptomic comparison between these species has shown a conservation of core inflammatory programs in various inflammatory contexts (7). Remarkably, our findings reveal ∼25% more protein identifications in mouse than in human neutrophils. Our imaging data and protein estimation assays confirm that human neutrophils are significantly larger and hence have a bigger protein amount than mouse cells. Hence, murine neutrophils translate more genes than their human counterparts. Recently, Sollberger *et. al*. also reported ∼11% more protein identifications in mouse than in human blood neutrophils (37). Our bigger observed difference suggests that some of these proteins identified as mouse-specific in our study might be present in humans at low abundance, falling below the detection limit in our analysis of 1000 cells. However, both studies confirm the enrichment of granule proteins in human neutrophils and of proteins associated with cell cycle, RNA metabolism, transport, and translation in murine neutrophils. The higher mass-specific metabolic rates and reactive oxygen species (ROS) production, faster cell cycle, and differences in cellular senescence in mice than in humans may influence their protein profiles (46, 80). The excess mitochondrial proteins detected in murine neutrophils emphasizes the species-specific differences in mitochondrial networking and the overall complexity, resembling morphological differences between human and mouse mitochondria in muscle fibers (81). Our analysis showed that all proteins with over half a million copies together accounted for 86% of total protein abundance in humans compared to only 75% in mice. This indicates that mouse neutrophils would have more distinct proteins per unit volume than humans. Despite all differences, we find that 80% of the proteins identified in humans have an ortholog in mice. This is consistent with genome data, highlighting an 85% overlap in protein-coding regions (82). Additionally, there is a correlation (r = 0.79) between both species in terms of their protein copy numbers which appears to be mirrored on the transcriptional level (7).

Our approach allowed to demonstrate a distinct protein expression pattern between brain infiltrated and circulating neutrophils 24 h post stroke. Recently it was shown that brain-infiltrating neutrophils in stroke were continuously recruited from blood and not differentiated locally (83). It will thus be interesting to study neutrophil proteomes at different time-points after stroke to see whether the initial activated phenotype is maintained or gradually reverted. Furthermore, atypical, transcriptionally immature neutrophils were found in aged mice after stroke, aggravating stroke pathology, in which CD101^-^ cells were the key regulators (61). Our proteomics analysis shows the downregulation of CD101 also in young mice, indicating a broader role of such neutrophil phenotypes in stroke pathophysiology.

Interestingly, our analysis showed an increase in proteins associated with the mitochondrial TCA cycle, fatty acid metabolism, and ETC in brain-derived neutrophils. The role of mitochondrial dysfunction and excessive oxidative stress on post-stroke consequences has been studied (84). Mitochondria in neutrophils play a remarkable role in mediating multiple functions including migration, metabolism, ROS production, degranulation, and neutrophil extracellular trap formation (85). The observed upregulation of mitochondrial TCA cycle in our study facilitates the energy metabolism in neutrophils to prolong their survival in glucose-deprived condition. These adaptations might be in alignment with CD101^-^ neutrophil response to glucose-deprived conditions (63). Furthermore, we identified differential regulation of respiratory electron transport chain components in neutrophils following stroke. Hypoxia is a common occurrence following stroke which exacerbates ischemic brain damage. Such hypoxic conditions can intensify a neutrophil-mediated inflammatory response (86). This leads to elevated levels of degranulation and NET release which were observed to peak 3 to 5 days post-stroke (87). Although hypoxia interferes with respiration (64), the increased production of ETC proteins in brain-derived neutrophils compared to circulating one after 24h post stroke may serves as a protective strategy to augment the ATP production and prolong their survival under oxygen-deprived condition. Interestingly, we identified differential regulation of proteins complex I, complex III, cytochrome b5 reductase(Cyb5r3), monoamine oxidase (Maoa and Maob), and glycerol-3 phosphate dehydrogenase (Gpd2), which are known contributors to mitochondrial ROS production (88). This neutrophil-generated ROS may serve as a critical nexus-linking cell survival and neuroprotection with the stroke pathogenesis as shown in various models (89). Concurrently, the upregulation of antioxidants in these neutrophils may represent a protective countermeasure against oxidative stress. While our study provides initial insights into the activity of these neutrophils, a more comprehensive investigation into their mitochondrial function will be essential to elucidate the underlying mechanistic pathways in greater detail. Interestingly, we found that most of the neutrophil effector molecules involved in activation or degranulation are rather upregulated in circulatory neutrophils. This finding supports our recent study showing early activation of and NET-release by circulatory neutrophils 6 h after stroke, indicating a sustained activation and migration of blood neutrophils also 24 h after stroke (59). Moreover, we also showed increased levels of NET markers in the plasma of patients 24 h-72 h after stroke onset (59). In this regard, our results align well with transcriptional characterizations of circulatory and brain-derived neutrophils (83).

Our method can capture molecular alterations also in transmigrated human neutrophils. While it is quite striking that a normal food flavor recruits large numbers of immune cells to the oral cavity (66). Our analysis also demonstrates a functional shift of these transmigrated neutrophils in comparison to the circulating counterparts. Blood neutrophils show more inflammatory capacity indicated by the upregulation of processes involving “inflammatory response,” “cell migration,” and “adhesion”. This could indicate that neutrophils which transmigrate to the oral cavity undergo a “disarming” (90) to prevent an excessive immune response in the target organ. It stands to reason that the body ensures food intake does not trigger overwhelming immune reactions. Interestingly, the downregulation of CD101 in transmigrated neutrophils warrants further investigation into the role of the CD101 low/CD101- population in inflammation. Additionally, we found that oral cavity neutrophils display a higher phagocytic signature, likely because they suddenly get in contact with large numbers of microorganisms. Our proteomic analysis of the oral cavity and blood neutrophils revealed a surprising number of proteins that were only detected in the oral cavity including different keratins. While these findings have to be investigated further, we speculate that these proteins comprise an “acquired” proteome of the neutrophils which depends on the tissue environment, which has been shown to play a role in physiological processes as tissue repair (91). This “acquired” proteome could play a role in functional neutrophil diversity in different organs which have been reported previously using different transcriptomic and functional approaches (13). Our work should thus trigger a re-evaluation of the impact of physiological processes like food uptake on neutrophil biology and provides a technology to investigate the impact of other food and liquid components on immune cells.

In conclusion, our study not only advances the methodologies for analyzing limited sample materials but also contributes significant insights into the proteomic distinctions between human and mouse neutrophils, both under physiological conditions and in response to sterile inflammation. While it is possible to obtain more than 1000 neutrophils from murine ischemic brain or the human oral cavity, our protocol establishes a foundation for future human studies with a focus on samples where the maximum cell count may be limited to 1000 cells, such as tumor-infiltrating neutrophils. This work paves the way for more in-depth investigations into the roles of neutrophils in various contexts, ultimately advancing our understanding of neutrophil-driven immune processes. Although our method enables the differentiation of neutrophil subpopulations among different residing or inflammatory sites based on their proteome, it may have limitations in distinguishing subpopulations within a single inflammatory site. Therefore, in the future, it might even be possible to elucidate proteomes from single neutrophil or their granular contents, thereby facilitating studies comparing neutrophil subpopulations and their kinetics in disease biology.

## Data Availability

The mass spectrometry proteomics data have been deposited to the ProteomeXchange Consortium *via* the PRIDE (92) partner repository with the dataset identifier PXD050682 (Username: reviewer_pxd050682@ebi.ac.uk, Password: iMDtTuGV). All data are publicly available as of the date of publication. Code used to generate interactive plot have been deposited to GitHub https://zenodo.org/doi/10.5281/zenodo.10817746. Additional information required to reanalyze the data will be shared by lead contact upon request.

## Supplemental data

This article contains supplemental data (36, 37).

## Conflicts of interests

The authors declare no competing interests.

## Supplementary Data

Table S6

DIA-Checklist_SG

Clinical-Checklist_SG_paper_AAT

Copy number plot_Neutrophil
