# Supplementary material for: Proteomic Characterization of 1000 Human and Murine Neutrophils Freshly Isolated From Blood and Sites of Sterile Inflammation
Source: Mol Cell Proteomics. 2024 Oct 11;23(11):100858. doi: 10.1016/j.mcpro.2024.100858 (PMC11630641; doi:10.1016/j.mcpro.2024.100858)
Supplement: Supplementary figure 1 [file mmc1.pdf]

Supplementary figure 1

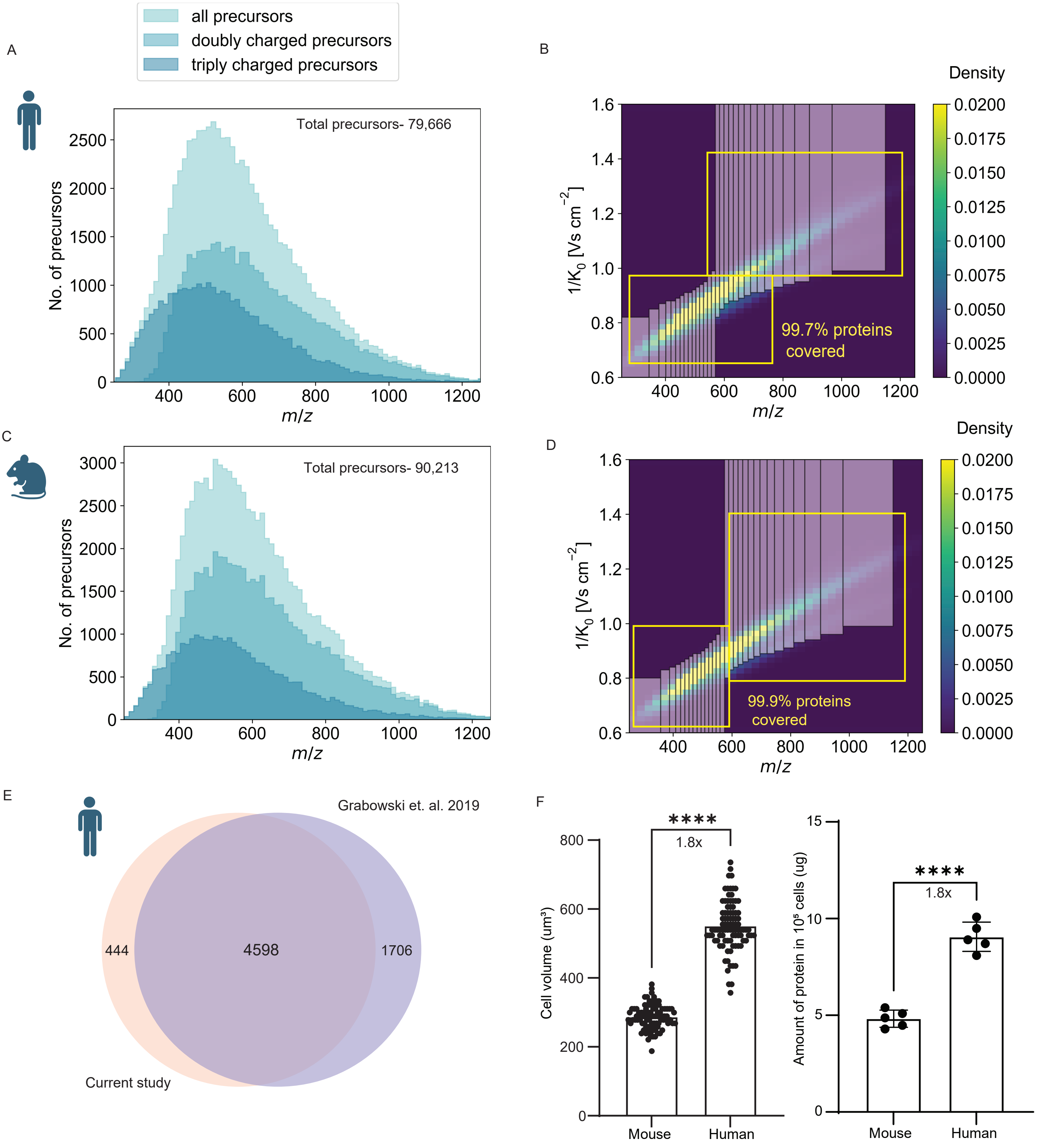

Figure S1: Species-specific spectral library proteome coverage. (A, C) Distribution of precursor charge states within organism-specific spectral libraries. (B, D) Distribution of precursors across the ion mobility-mass-to-charge (IM-m/z) plane covered by our optimized analytical method. (E) Venn diagram representing the overlap of human neutrophil proteome spectral library in our current study and repository data 1. (F) Cell volume and protein content of individual neutrophil in human and mouse. Data represent mean ± s.d., ****p<0.0001
