# Supplementary material for: Proteomic Characterization of 1000 Human and Murine Neutrophils Freshly Isolated From Blood and Sites of Sterile Inflammation
Source: Mol Cell Proteomics. 2024 Oct 11;23(11):100858. doi: 10.1016/j.mcpro.2024.100858 (PMC11630641; doi:10.1016/j.mcpro.2024.100858)
Supplement: Supplementary figure 2 [file mmc2.pdf]

Supplementary figure 2

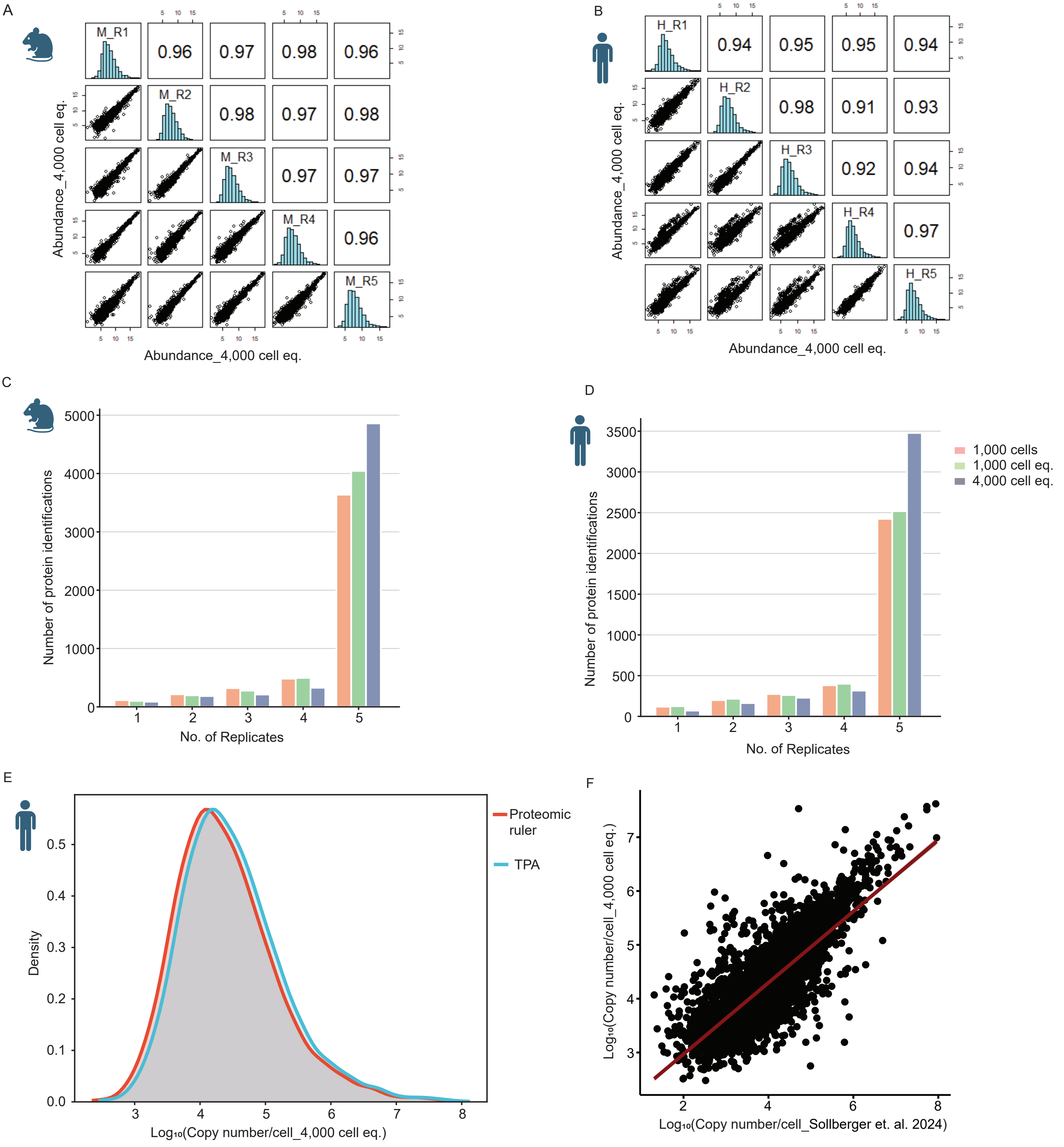

Figure S2: Method reproducibility. (A, B) Proteins identified in one or more than one replicates of resting circulatory neutrophils in both organisms. (C, D) Irrespective of the organisms, the biological replicates exhibited strong correlation (r > 0.9) based on their abundances. (E) A uniform trend is observed in the copy number distribution of 1,000 cells as estimated using both the Proteomic Ruler approach as well as the Total Protein Approach (TPA). (F) Correlation between current study and repository data 2 in estimating mouse protein copies.
