# Supplementary material for: Proteomic Characterization of 1000 Human and Murine Neutrophils Freshly Isolated From Blood and Sites of Sterile Inflammation
Source: Mol Cell Proteomics. 2024 Oct 11;23(11):100858. doi: 10.1016/j.mcpro.2024.100858 (PMC11630641; doi:10.1016/j.mcpro.2024.100858)
Supplement: Supplementary figure 3 [file mmc3.pdf]

Supplementary figure 3

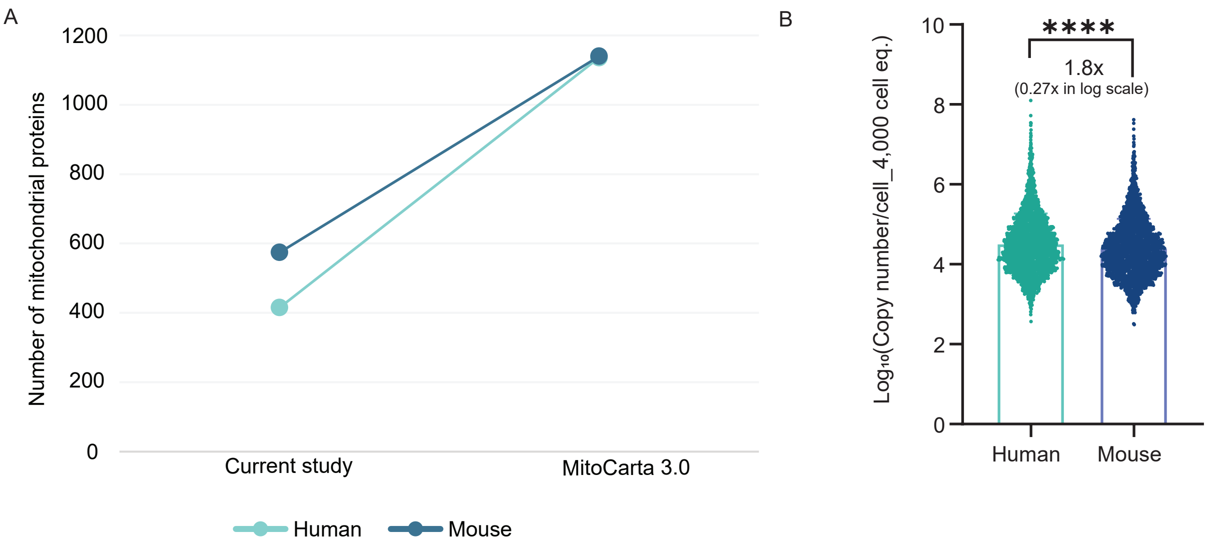

Figure S3: Divergence in human and mouse neutrophil. (A) Differences in mitochondrial protein identification in mouse and human neutrophils (4,000 cell eq. proteome data) in relation to MitoCarta database. (B) Bar plot showing the copy number of shared proteins between human and mouse. Each dot represents the average copy number of individual proteins from biological replicates.
