# Supplementary material for: Proteomic Characterization of 1000 Human and Murine Neutrophils Freshly Isolated From Blood and Sites of Sterile Inflammation
Source: Mol Cell Proteomics. 2024 Oct 11;23(11):100858. doi: 10.1016/j.mcpro.2024.100858 (PMC11630641; doi:10.1016/j.mcpro.2024.100858)
Supplement: Supplementary figure 4 [file mmc4.pdf]

Supplementary figure 4

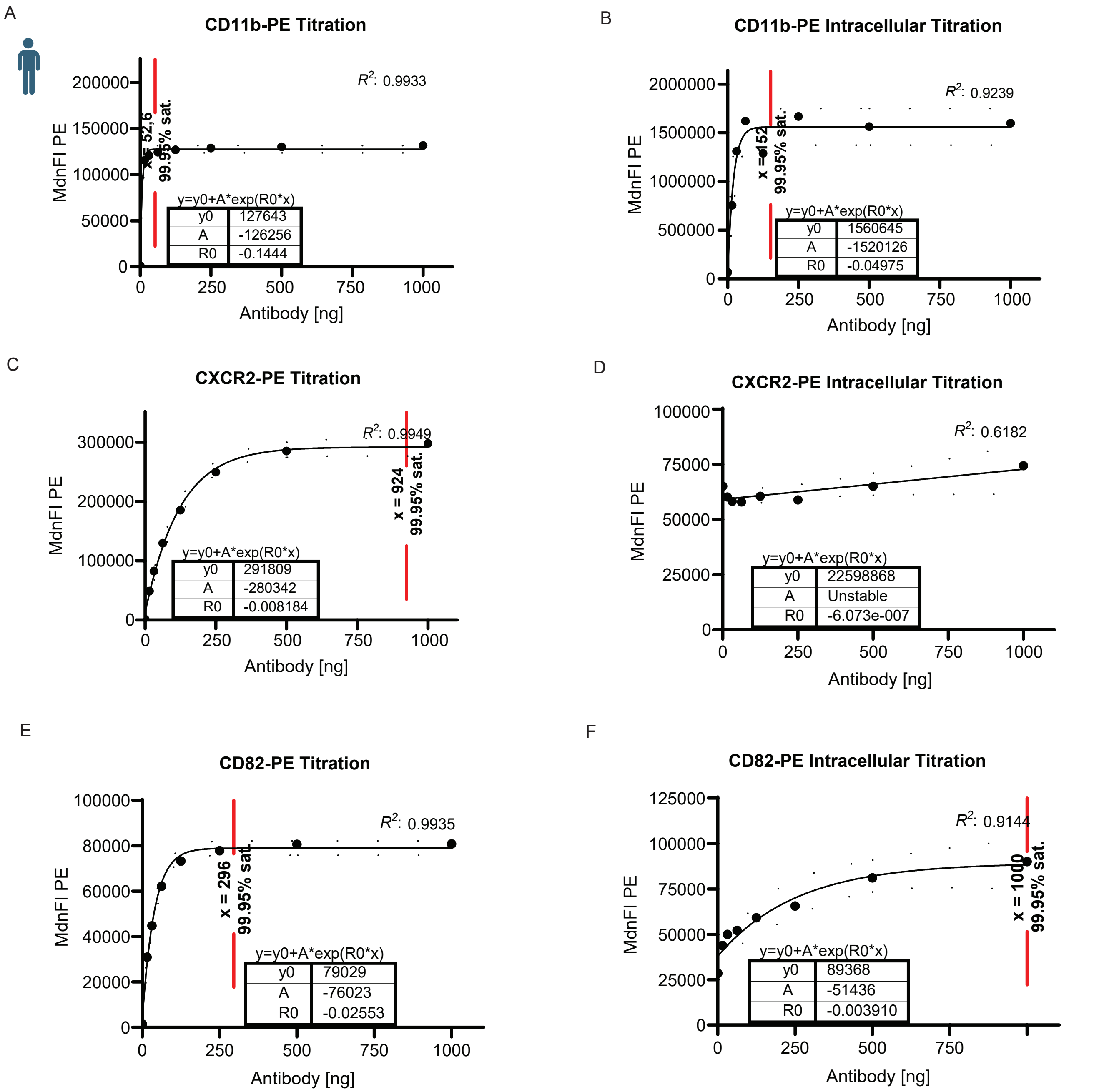

Figure S4: Saturation curves of human antibodies used in qFlow measurement. Titration curves used to quantify human neutrophil proteins for both surface (A, C, E) as well as surface and intracellular portions after fixation and permeabilization (B, D, F).
