# Supplementary material for: Proteomic Characterization of 1000 Human and Murine Neutrophils Freshly Isolated From Blood and Sites of Sterile Inflammation
Source: Mol Cell Proteomics. 2024 Oct 11;23(11):100858. doi: 10.1016/j.mcpro.2024.100858 (PMC11630641; doi:10.1016/j.mcpro.2024.100858)
Supplement: Supplementary figure 5 [file mmc5.pdf]

Supplementary figure 5

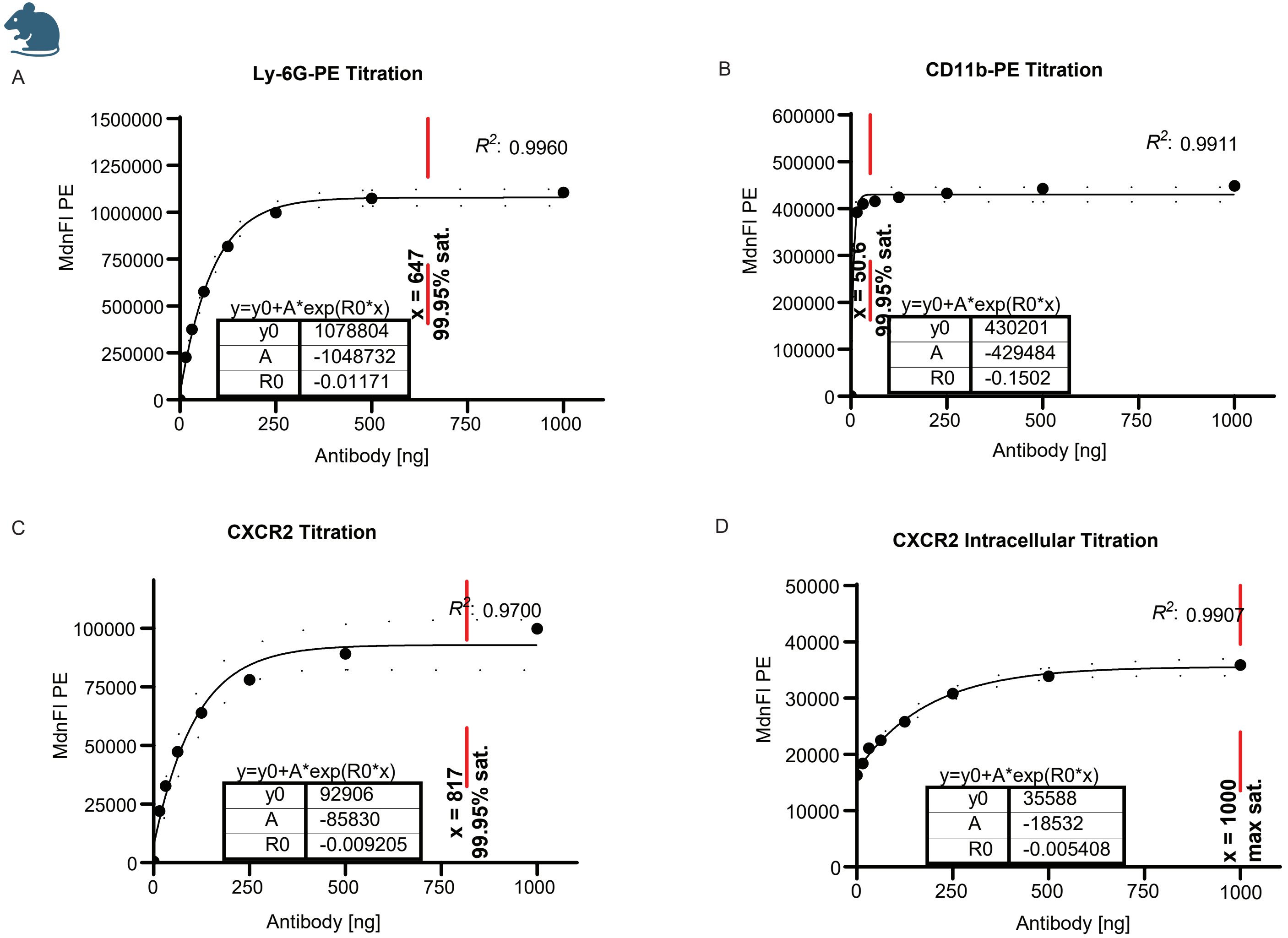

Figure S5: Saturation curves of mouse antibodies used in qFlow measurement. Titration curves of antibodies used to quantify mouse neutrophil proteins for both surface as well as surface and intracellular portions after fixation and permeabilization.
