# Supplementary material for: Proteomic Characterization of 1000 Human and Murine Neutrophils Freshly Isolated From Blood and Sites of Sterile Inflammation
Source: Mol Cell Proteomics. 2024 Oct 11;23(11):100858. doi: 10.1016/j.mcpro.2024.100858 (PMC11630641; doi:10.1016/j.mcpro.2024.100858)
Supplement: Supplementary figure 6-1 [file mmc6.pdf]

Supplementary figure 6

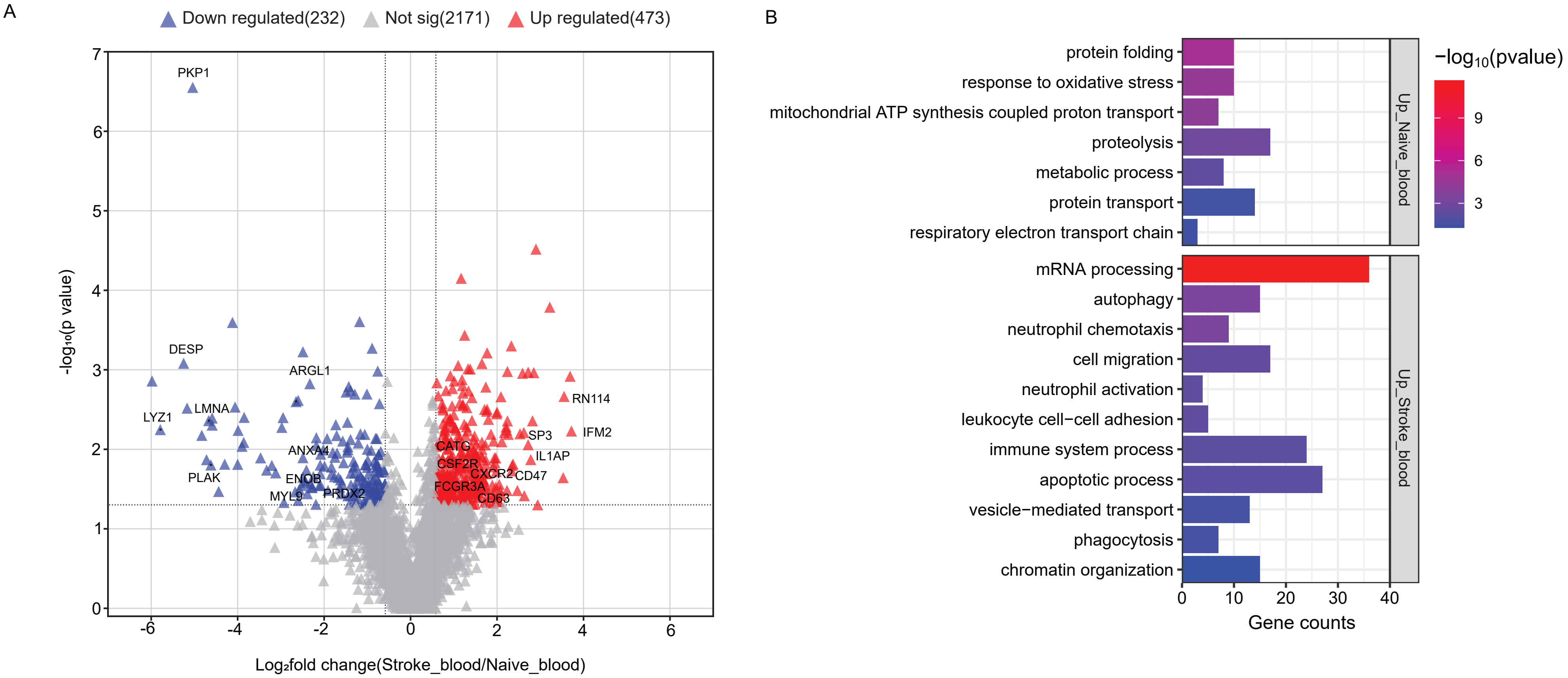

Figure S6: Differential protein expression in circulating neutrophils 24h post stroke. A. Volcano plot showing the proteins upregulated (red) or downregulated (blue) protein in neutrophils derived from stroke mice compared to naive mice (n=3). Statistical significance was determined by paired t-test with p-value <0.05 and log2fold change ≥ ±0.58. B. Biological processes (Benjamini-Hochberg adjusted p-value <0.05) associated with dysregulated proteins in stroke mice.
