# Supplementary material for: Proteomic Characterization of 1000 Human and Murine Neutrophils Freshly Isolated From Blood and Sites of Sterile Inflammation
Source: Mol Cell Proteomics. 2024 Oct 11;23(11):100858. doi: 10.1016/j.mcpro.2024.100858 (PMC11630641; doi:10.1016/j.mcpro.2024.100858)
Supplement: DIA-Checklist_SG [file mmc13.pdf]

MCP MOLECULAR & CELLULAR PROTEOMICS

Checklist for Publication of Data Independent Acquisition (DIA) Data in MCP

As there are many different strategies for analyzing DIA data, it is expected that not all of the guidelines below will be relevant to a given analysis, so authors should adhere to the guidelines appropriate to their approach. The checklist below is to assist authors in making sure they have provided the required information, but the full DIA guidelines contain more detail, so there is required information not explicitly stated in this checklist. Hence, authors of DIA studies should read the full guidelines.

The following information must be included in the *Experimental Procedures* section:

Experimental Design and Statistical Rationale:

			Page
Authors provided a subsection in the *Experimental Procedures* section with the header "*Experimental Design and Statistical Rationale*" that contains the following information:	Y	N/A	12
The numbers and types of sample conditions employed	Y	N/A	12
The methods used for sample acquisition order randomization (if appropriate for your study)	Y	N/A	
If retention time standards or other spiked protein or peptide standards were used	Y	N/A	12
Whether MS1 data was acquired	Y	N/A	7
Whether m/z range was fractionated for fragmentation analysis; and if so, parameter of separation (m/z, mobility), number of windows, whether overlapping windows were acquired, and total cycle time.	Y	N/A	7
If a library was created as part of the work for subsequent peptide detection, the number and type (biological/technical) of samples used for library creation	Y	N/A	6,12
Description or reference to the statistical tests used for subsequent data analyses	Y	N/A	12
Justification for choice of statistical tests used.	Y	N/A	

For Spectrum-Centric DIA Analysis:

Peak List Generation:

			Page
Software used to create peak lists	Y	N/A	
Parameters used in peak list generation, such as thresholding, de-multiplexing, de-isotoping	Y	N/A	
Definition of how retention/drift time and intensity were assigned to peaks	Y	N/A	
State the maximum number of precursor peak lists an observed fragment ion could be included in	Y	N/A	

Search Engine:

			Page
The name and version of software used for database searching	Y	N/A	
The name and release version/date of all sequence databases or spectral libraries used.	Y	N/A	

If library generated in-house, source of sequences and software used to compile it	Y	N/A	
The number of entries searched and the number of proteins these entries cover	Y	N/A	
Enzyme specificity, including number of missed cleavages and non-specific cleavages allowed	Y	N/A	
List of all fixed modification(s) (including residue specificity) considered	Y	N/A	
List of all variable modification(s) (including residue specificity) considered	Y	N/A	
Mass tolerance for precursor (if relevant) and fragment ions	Y	N/A	
Threshold score/expectation value for accepting individual spectra	Y	N/A	
Estimated False Discovery Rate (FDR) at peptide, protein or batch level for reported results	Y	N/A	
Description of how this FDR was calculated	Y	N/A	

For Peptide-Centric DIA Analysis:

Spectral Library:

			Page
Source of library is stated	Y	N/A	6
If created as part of this study, DDA MS/MS guidelines have been completed	Y	N/A	7
Software used for library generation is stated, including all relevant parameters employed such as how multiple spectra of the same precursor were handled, and whether any thresholding / removal of peaks were performed	Y	N/A	7
Estimated FDR of entries in library, including method of estimation employed	Y	N/A	7
If public library used, version/release number and location it can be downloaded from	Y	N/A	
Number of entries searched, including number of proteins these correspond to	Y	N/A	7
Whether additional metadata such as retention time or ion mobility was associated with spectra	Y	N/A	
If decoy entries were created, how were they created, how many of these were searched, and how were these assigned to proteins to allow protein-level FDR estimation (if relevant)	Y	N/A	

Matching of Data to Spectral Library:

Name and version number of software used for searching	Y	N/A	7
If precursor ion detection was attempted, how this was used and mass tolerance employed for matching	Y	N/A	8
If retention time or ion mobility was used, what tolerances were applied and how these were aligned between acquisitions	Y	N/A	8

How many peaks were considered for identifying peptides, and how these were selected (intensity; within a certain mass range)	Y	N/A	8
Mass tolerance for matching between library and experimental data	Y	N/A	
If modifications are reported, how site localization was evaluated	Y	N/A	
Estimated FDR at peptide, protein and/or batch level, including how this was calculated.	Y	N/A	8

The following information must be included in the *Results* or *Supplemental* sections:

**For results reported at the protein level, the results table must include:**			**Page**
Protein accession number			
Number of distinct peptide sequences assigned to a protein	Y	N/A	
If identified by library searching, number of distinct peptides from this protein in the library	Y	N/A	
For proteins identified by a single peptide, annotated spectra or chromatograms are provided	Y	N/A	

**For results reported at the peptide level the results table must include:**			
Protein accession number	Y	N/A	
All peptide sequences assigned, including all modifications	Y	N/A	
Observed precursor m/z and z (if MS1 data used)	Y	N/A	
For spectrum-centric analysis the score and/or statistical measure associated with the peptide identification	Y	N/A	
If a biological modification is reported, measure of reliability for site localization, or indication of ambiguity	Y	N/A	
For peptides bearing a biological modification or proteins identified based on a single peptide sequence annotated spectra or chromatograms are provided	Y	N/A	

**Quantification**			**Page**
Quantification measures in the peptide or protein results table, along with description of how these values were derived from the raw data	Y	N/A	
Description of post-processing steps such as outlier rejection, minimum intensity thresholds	Y	N/A	10
Number of peptides used for quantification (if different from number used for identification)	Y	N/A	10
Description of statistics used to measure reliability/reproducibility of measurements at technical and biological levels	Y	N/A	10

Description of how isoforms in a protein group were quantified (if relevant) Y N/A

Raw Data Submission

The raw mass spectrometric data has been deposited **Y**

The location and identifying information (URL of repository, deposit ID, username, hash code/identifier, password) are:

Note: The reviewers' login information (username, password) to access the raw data also needs to be included in the cover letter

The mass spectrometry proteomics data have been deposited to th
