# Supplementary material for: Proteomic Characterization of 1000 Human and Murine Neutrophils Freshly Isolated From Blood and Sites of Sterile Inflammation
Source: Mol Cell Proteomics. 2024 Oct 11;23(11):100858. doi: 10.1016/j.mcpro.2024.100858 (PMC11630641; doi:10.1016/j.mcpro.2024.100858)
Supplement: Clinical-Checklist_SG_paper_AAT [file mmc14.pdf]

MCP MOLECULAR & CELLULAR PROTEOMICS

Checklist for Preparing Manuscripts Describing Research in Clinical Proteomics

To aid authors in preparing their manuscripts, the check list of the items that must be included is given below. Authors are encouraged to print out this document and use it to ascertain that the complete set of required information has been included. Click here to open a set of guidelines that explains each point on the list, and why it has been included. Authors submitting this type of data for the first time are particularly encouraged to use both the checklist and guidelines.

1. Ethics Approvals

			Page
Statement of institutional Ethics/Animal Committee (IRB/ACUC) approval is included in the text of the article. **(This is required of all articles of this type without exception)**	Y		28

2. Study Goals and Design

A comprehensive description is provided that includes the following:

			Page
The stage/phase of the study (discovery, verification or validation) and the stage/phase that a candidate (biomarker, target, analyte) is at (exploratory/discovery; preclinical validation; etc.) is stated	Y	N/A X	
The flow of subjects/samples through the study, including the initial number of cases, the number included in each stage of the analysis (a diagram is recommended for more complex/larger studies) and reasons for subject dropout are provided	Y	N/A X	

3. Subject Sources and Description

The source and classification of subjects (or materials obtained there from) are described with respect to as many of the following parameters as are known

			Page
The source of biospecimens (e.g. centralized biobank, internal biobank, clinical trial collection, surgery etc) are described	Y	N/A	28
Inclusion/exclusion criteria for the study and reference cohorts are given.	Y	N/A X	

For all patients from whom samples were derived, a definition of the disease is given that includes:

A disease description, such as subtype, stage, grade, histology and clinical score (if applicable)	Y	N/A X	
Disease type using standard medical terminology (include ICD codes where known), e.g. juvenile diabetes v. noninsulin-dependent diabetes.	Y	N/A X	
Any known potential confounders relative to the time of sampling, such as intra- or pre-operative status, administered drugs/anesthetics etc	Y	N/A X	
Intrinsic factors, demographic and clinical characteristics for study and reference (control) subjects, including age gender, disease stage, and co-morbidities etc are provided	Y	N/A	28

MCP MOLECULAR & CELLULAR PROTEOMICS

4. Biospecimen Qualifications

Tissue

			Page
The type of processing, e.g. formalin, ethanol, embedding medium, method of freezing, lysis solution etc.	Y	N/A	
The post-cutting fixation for frozen tissue	Y	N/A	
The methods of enrichment for relevant component(s) of biospeciman if applicable (e.g. micro dissection, fractionation)	Y	N/A	
Any histological review of biospecimens used in the reported experiments	Y	N/A	

Biofluids

For blood and biological fluid biospecimens, standard operating procedures, if used, are referenced	Y	N/A	

If no, the following information was provided:

The method of collection. [In the case of urine samples, the collection mode should be stated: 24-h, first morning, second morning or random urine early stream or midstream.]	Y	N/A	28
The tube type (and size, if known) used for collection and storage	Y	N/A	28
The use of additives such as anti-coagulants, preservatives, and protease inhibitors, if used	Y	N/A	28
The processing conditions including the time interval between collection and separation, centrifugation conditions, temperature of processing, time interval between processing and freezing	Y	N/A	28

Primary cell lines

For primary cell lines, generation and use, the following information is provided:

Clinical details regarding subject and biospecimen of origin	Y	N/A	
The conditions/protocols of cell line generation and characterization including passage number and number of clones analyzed.	Y	N/A	

5. Statistical Considerations

The statistical analysis strategy is described in detail that should include:

			Page
The central hypothesis that is being tested	Y	N/A	
Model building and validation	Y	N/A	
A clear definition of the statistical algorithm used	Y	N/A	

The rationale used to choose cut-off thresholds and other model parameters	Y	N/A	
The independence of exploratory (training) and confirmative (test) analyses	Y	N/A	

The following details about the statistical analyses are provided:

Point estimates, p-values and/or confidence intervals	Y	N/A	

6. Technical Considerations

			Page
The performance characteristics (technical and process including fractionation, digestion etc.) of the analytical process/assay(s) used (e.g. mass spectrometry, protein, antibody, nucleic acid arrays, immuno-chemistry, 2D gel electrophoresis, or other measurement technology) must be described in the Experimental Procedures section of the manuscript. Identification of peptides, proteins and post-translational modifications by mass spectrometry must meet the separate criteria for such articles.	Y	N/A	
