# Supplementary material for: Proteomic Characterization of 1000 Human and Murine Neutrophils Freshly Isolated From Blood and Sites of Sterile Inflammation
Source: Mol Cell Proteomics. 2024 Oct 11;23(11):100858. doi: 10.1016/j.mcpro.2024.100858 (PMC11630641; doi:10.1016/j.mcpro.2024.100858)
Supplement: Copy number plot_Neutrophil [file mmc15.zip › mcpro_100858_Copy number plot_Neutrophil_mmc15.html]

Human and Mouse\_copy number per cell (4,000 cell eq.)
